# Mechanisms of Collagen Crosslinking in Diabetes and Keratoconus

**DOI:** 10.3390/cells8101239

**Published:** 2019-10-11

**Authors:** Tina B. McKay, Shrestha Priyadarsini, Dimitrios Karamichos

**Affiliations:** 1 Schepens Eye Research Institute/Massachusetts Eye and Ear, Department of Ophthalmology, Harvard Medical School, Boston, MA 02114, USA; tina_mckay@meei.harvard.edu; 2 Department of Ophthalmology/Dean McGee Eye Institute, University of Oklahoma Health Science Center, Oklahoma City, OK 73104, USA; shrestha.priyadarsini@gmail.com

**Keywords:** cornea, crosslinking, diabetes, keratoconus, collagen, riboflavin, lysyl oxidase, advanced glycation end products

## Abstract

Collagen crosslinking provides the mechanical strength required for physiological maintenance of the extracellular matrix in most tissues in the human body, including the cornea. Aging and diabetes mellitus (DM) are processes that are both associated with increased collagen crosslinking that leads to increased corneal rigidity. By contrast, keratoconus (KC) is a corneal thinning disease associated with decreased mechanical stiffness leading to ectasia of the central cornea. Studies have suggested that crosslinking mediated by reactive advanced glycation end products during DM may protect the cornea from KC development. Parallel to this hypothesis, riboflavin-mediated photoreactive corneal crosslinking has been proposed as a therapeutic option to halt the progression of corneal thinning by inducing intra- and intermolecular crosslink formation within the collagen fibrils of the stroma, leading to stabilization of the disease. Here, we review the pathobiology of DM and KC in the context of corneal structure, the epidemiology behind the inverse correlation of DM and KC development, and the chemical mechanisms of lysyl oxidase-mediated crosslinking, advanced glycation end product-mediated crosslinking, and photoreactive riboflavin-mediated corneal crosslinking. The goal of this review is to define the biological and chemical pathways important in physiological and pathological processes related to collagen crosslinking in DM and KC.

## 1. Introduction

Collagen is the most abundant protein in the cornea and comprises roughly one-third of total protein content in the human body [1]. The expression and organization of collagen and the extracellular matrix (ECM) within the cornea are highly regulated processes coordinated to maintain the structural, mechanical, and refractive properties of the tissue. The corneal stroma comprises over 90% of the thickness of the cornea and contributes up to two-thirds of the refractive power of the eye, as well as serves as a protective barrier against the external environment to prevent injury or infection. More than 20 distinct collagen types have been identified with varying isoform distributions found throughout the body [1]. Tissue-dependent expression of specific collagen isoforms and proteoglycans influence a tissue’s biomechanical properties, i.e., stiffness and elasticity, based on fibril size and organization. The dominant collagen isoforms present in the human corneal stroma are heterotypic fibrils of collagen types I and V [2] and small amounts of types VI, XII, XIII, and XIV, among others [3,4]. Descemet’s membrane is predominately composed of type VIII collagen [5] with the epithelial and endothelial basement membranes composed of type IV collagen [6].

The collagen structure within the cornea is organized in a hierarchical manner, beginning with pro-collagen monomers that are cleaved to generate tropocollagen (length = 300 nm and diameter = 1.5 nm), which crosslinks into small bundles to form microfibrils (length = 4–12 μm and diameter = 20 nm) that then elongate to form a collagen fibril (diameter = 30 nm) [4,7]. Multiple tissue-specific mechanisms are present to control ECM deposition within the cornea, including at the level of collagen transcription to post-translational modifications and secretion as well as post-secretory processing and crosslinking.

**Figure 1:**
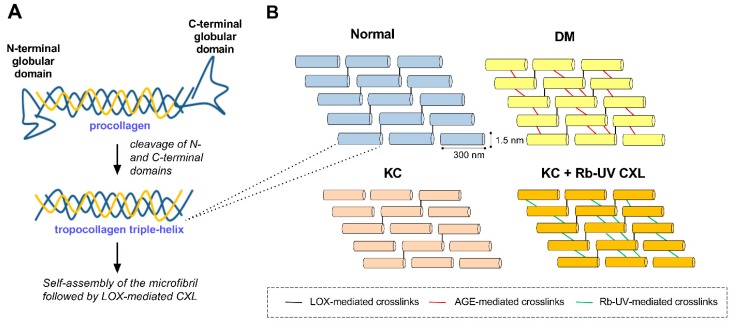
Schematic depicting fibrillar collagen processing and crosslinking in normal, diabetes mellitus (DM), and keratoconus (KC) microenvironments. (**A**) Pro-collagen secretion, cleavage, and self-assembly of tropocollagen to form collagen fibrils. (**B**) Relative distribution of collagen crosslinks presents within normal, DM, and KC corneas. Lysyl oxidase (LOX) catalyzes formation of covalent bonds between lysine or hydroxylysine groups from the C-terminus of one molecule to the N-terminus of an adjacent molecule (black lines). DM has been associated with increased crosslinks mediated by advanced glycation end products (AGEs) (red lines), while KC has been linked to an overall reduction in LOX-mediated crosslinks. Photooxidative corneal crosslinking (CXL) mediated by riboflavin (Rb) is thought to increase the number of collagen crosslinks between fibers (green lines). These enzymatic (e.g., LOX) and non-enzymatic reactions (e.g., AGE- and Rb-UV-mediated) promote the formation of inter- and intramolecular crosslinks between the helical domains of tropocollagen that lead to increased tissue stiffness.

Collagen is secreted from the corneal keratocytes as an inactive triple-helical pro-collagen molecule containing inhibitory globular domains that prevent fibril self-assembly (Figure 1). Upon cleavage of the N- and C-terminal ends by extracellular proteinases, ADAMTS, and BMP1/tolloid-like proteins, respectively, tropocollagen self-assembles into cross-striated microfibrils predominately composed of type I and V collagens [4,8]. Type V collagen maintains its bulky N-terminal globular domain in the collagen fibrils of the cornea, thereby serving to limit fibril diameter presumably via steric hindrance by preventing further binding of type I collagen monomers [9]. The addition of covalent bonds between adjacent tropocollagen molecules by lysyl oxidase further stabilizes the organization and structure of these microfibrils as they organize end-to-end laterally and coil together to form mature fibrils. A mature fibril is composed of ~70 microfibrils consisting of type I and V collagens at a ratio of 10:1 [4,10]. Small leucine-rich proteoglycans, e.g., keratocan, lumican, decorin, and mimecan (osteoglycin), are associated with secreted collagen and thereby influence the lateral growth and organization of fibrils [11]. Mature collagen fibrils organize according to an alternating orthogonal orientation into a lamellar structure within the corneal stroma consisting of ~300–500 layers parallel to the ocular surface. These fibrils enable sufficient corneal rigidity and elasticity to withstand mechanical stress, as well as allow for transparency of the tissue via strict organization and alignment of the stromal matrix.

Two diseases associated with corneal stromal defects include diabetes mellitus (DM) and keratoconus (KC). The underlying pathobiology involved in each disease is quite distinct, thus giving rise to pointed differences in collagen structure, leading to corneal thickening and thinning in DM and KC, respectively. While DM is associated with increased collagen crosslinking mediated by glycated proteins in situ, the FDA-approved photooxidative corneal crosslinking (CXL) using riboflavin for the treatment of KC also leads to stromal stiffening presumably by inducing crosslink formation between the α-helical domains of collagen protofibrils and possibly between fibrils themselves (Figure 1). The effects of these chemical modifications on collagen structure lead to biomechanical changes within the cornea, namely, decreased elasticity of the stroma. Induction of non-enzymatic crosslinks within the collagen matrix via advanced glycation end product (AGE) and photoreactive processes are thought to prevent central corneal thinning, giving rise to lower predominance of KC development in the DM population. As of now, the role of AGE-mediated crosslinking in KC prevention or stabilization has been posited based on epidemiological studies, and remains yet unproven. 

In this review, we describe the pathophysiology of DM and KC and their effects on ECM structure within the corneal stroma in the context of changes in cellular function and biomechanical properties, as well as current therapeutic options for each disease. Due to the narrative that DM patients may be protected from KC, we further discuss the chemical and biological processes involved in collagen crosslinking mediated via two non-enzymatic processes important in DM and the treatment of KC: (1) the Maillard reaction involving glucose and ECM proteins generating AGEs susceptible to crosslinking; and (2) photooxidative CXL mediated by riboflavin. Both chemical reactions modulate the biomechanical properties of the cornea by influencing the thickness and elasticity of the corneal stroma. We further compare the chemistry involved in these reactions to enzymatic crosslinking mediated by lysyl oxidase to describe parallel attributes in terms of collagen structure that may explain the inverse correlation of KC development in DM patients.

## 2. Biology of DM and KC

**Figure 2:**
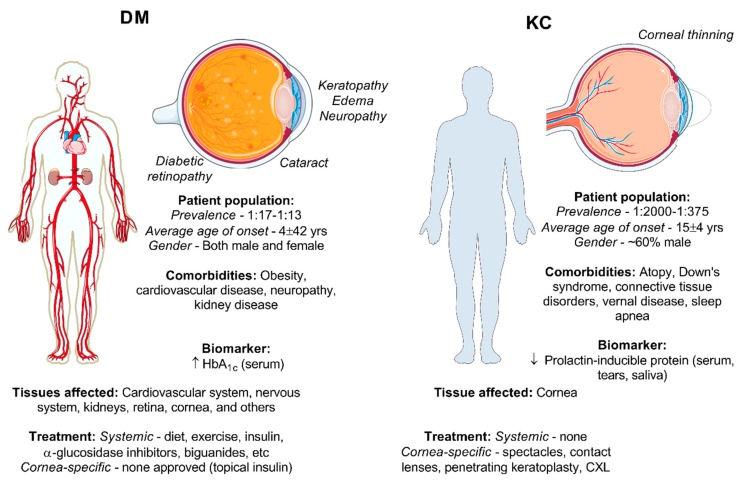
Comparison of the DM and KC condition based on the general patient population, systemic clinical features, ocular effects, and treatments for DM and KC. Pictorials modified from Medical Servier Art based on a Creative Commons Attribution 3.0 Unported License available at www.servier.com.

DM and KC have complex disease etiologies with contrasting features. DM and KC both present with defects in the cornea, albeit with opposing effects on collagen structure and, likewise, the underlying systemic characteristics are quite distinct (Figure 2). The patient population of DM is highly heterogeneous depending on the diagnosis of type 1 DM (T1DM), type 2 DM (T2DM), or gestational diabetes, with similar broad variability in the KC population making it difficult to understand the underlying genetic components. DM affects people of all ages from childhood to adulthood with the average age of onset ranging from 4 to 42 years old depending on whether T1DM or T2DM develops [12,13,14]. By contrast, KC is commonly diagnosed during puberty and often stabilizes in the third to fourth decade of life [15]. The rate of KC progression varies greatly between individuals; however, its incidence is usually higher in younger patients. DM is predominately a systemic condition linked to prolonged hyperglycemia leading to global effects on the body, including macrovascular and microvascular damage to the cardiovascular system, nervous system, kidneys, and retina. Interestingly, while KC is generally considered exclusively as a corneal disease, recent work has identified variations in key biomarkers associated with hormone levels in KC patients, suggesting that KC may also have a systemic component [16,17,18], though far less understood. In this section, we compare the general patient population of DM and KC, disease causation, systemic clinical features, and current treatments in order to understand the disease background found in each condition as to how they contribute to changes in the cornea. 

### 2.1. Pathophysiology of DM

DM is caused by autoimmune insulin depletion or acquired insulin resistance which give rise to chronic elevated blood glucose levels. Over 285 million people worldwide were reported to have DM in 2010, with estimates rising to 439 million people by 2030 [19]. The majority (90%) of cases are attributed to T2DM. The criteria for diagnosis of diabetes has been defined by the American Diabetes Association as the following [20]: (1) fasting plasma glucose level >126 mg/dL (7 mmol/L), (2) 2 h plasma glucose level following insulin challenge with 75 g of glucose >200 mg/dL (11.1 mmol/L), (3) HbA_1C_ > 6.5% (48 mmol/L), or (4) random plasma glucose level >200 mg/dL (11.1 mmol/L). These metrics may vary depending on ethnicity with minority groups in the United States, e.g., African Americans, Hispanic, Native Americans, and Asians, with impaired glucose tolerance showing higher HbA_1C_ levels compared to Caucasians independent of age, sex, and BMI, among other factors [21]. In the United States, Native Americans show the highest prevalence of DM (33%) compared to non-Hispanic Caucasians (7.1%) likely attributed to a higher genetic predisposition for DM development [22]. The patient population is diverse, affecting both males and females with similar T1DM and T2DM complications in advanced stages. Differences in severity of the disease or secondary complications based on sex have been reported [23], yet remain an area where relatively little is known. The average age of onset of T2DM is 42 years old, but may vary significantly depending on secondary complications and access to healthcare [12]. The average age of people with DM in developing countries based on the United Nations classification system is 40–60 years old compared to an older DM population (>60 years old) in developed countries [19] suggesting significant age disparities based on location and economic status.

In terms of disease development, T1DM is caused by an autoimmune-mediated loss of β-cells in the pancreas, leading to insufficient insulin production [20]. T1DM makes up about 10% of total DM cases and is commonly diagnosed in childhood at age 4 or older but may arise at any age. The presence of auto-antibodies targeting various auto-antigens, including glutamic acid decarboxylase and tyrosine phosphatases IA-2, are common biomarkers found in the serum of T1DM patients [24,25,26]. Consistent with a pro-inflammatory environment, T1DM has been associated with the development of other autoimmune disorders, including Grave’s disease and celiac disease, suggesting that shared genetic or nutritional factors may increase susceptibility to DM development [27,28]. 

By contrast, T2DM is caused by insulin resistance that often presents with deficient β-cell insulin secretion due to an inability to overcome persistently elevated blood glucose levels. Both T1DM and T2DM lead to hyperglycemia as a result of reduced glucose uptake from circulation by cells. The mechanisms underlying T2DM-induced damage are attributed to ‘chronic fuel surplus’, primarily in the form of excess glucose and fat, which contribute to visceral adipose tissue accumulation, insulin resistance, and pancreatic β-cell damage [29]. This condition thus gives rise to chronic hyperglycemia leading to the damage of several major organ systems, including the cardiovascular system and kidneys, as well as a number of ocular complications of the anterior and posterior segments. Extended periods of hyperglycemia promote the development of AGEs on proteins that are highly expressed, such as collagen, laminin, and heparan sulfate proteoglycans. These proteins tend to have a long-half life in the body, leading to a phenomenon termed ‘metabolic memory’, and may accumulate, leading to pathologies associated with vascular occlusion and endothelial damage [30,31,32]. Heritable genetic factors have been associated with the development of DM including key genes related to β-cell function and metabolism, such as transcription factor 7-like 2 (TCFL2), and fat mass and obesity-associated protein (FTO) [33]. A growing interest in the role of epigenetic modifications based on environmental factors, including diet and lifestyle, has focused on genetic determinants that may promote a predisposition to DM development [34].

### 2.2. Pathophysiology of KC

KC is a multifactorial, ectatic corneal thinning disease that leads to significant defects in visual acuity and development of irregular astigmatism. The prevalence has been reported to range from 1:2000 to 1:375 depending on the patient population [35,36] with an average age of onset in the second decade of life [37]. Diagnosis of KC with the Amsler–Krumeich classification system relies predominately on topography and thickness measurements of the cornea (e.g., curvature based on maximum keratometry (K_max_) and central corneal thickness (CCt_min_), respectively) with stages defined based on the following criteria: (1) Stage 1—Eccentric steepening, myopia and astigmatism <5 D, K_m_ < 48 D; (2) Stage 2—Myopia and astigmatism 5–8 D, K_m_ < 53 D and CCt_min_ > 400 μm; (3) Stage 3—Myopia and astigmatism 8–10 D, K_m_ > 53 D and CCt_min_ = 300–400 μm; (4) Stage 4—Refraction not measurable, K_m_ > 55 D and CCt_min_ < 200 μm, and scarring. The characteristic thinning of the central cornea contributes to scar development in the late stages, thereby requiring corneal transplantation to restore visual acuity.

Though the cause of KC remains elusive, a number of studies have suggested that reactive oxygen species (ROS) within the cornea due to exposure to external oxidants—such as UV light or intracellular ROS produced as byproducts of cellular metabolism—may drive pathological thinning of the stroma [38]. This focus on oxidative stress, or the inability of KC-derived stromal fibroblasts to withstand internal and external ROS, has been supported by metabolic studies identifying increased lactate production and lower reduced glutathione availability in KC-derived fibroblasts [39] and tear samples [40] compared to healthy controls. These studies are consistent in showing increased susceptibility to damage caused by cytosolic ROS [41] and hypoxia [42] likely attributed to reduced expression of superoxide dismutase in KC-derived fibroblasts [43] with genetic studies likewise identifying splice variants of SOD1 associated with KC in a human population [44,45,46]. Furthermore, studies have suggested defects in mitochondrial function based on the reduced expression of key mitochondrial genes, including SOD2 and PMAIP1 [47], consistent with altered metabolic flux favoring aerobic glycolysis in KC over the tricarboxylic acid cycle and oxidative phosphorylation for energy production [39]. To combat the increased oxidative stress exhibited in KC, studies utilizing the antioxidant quercetin have shown recovery of basal cellular metabolism in KC-derived fibroblasts with reduced lactate production similar to control levels [48], suggesting that therapeutics targeting the metabolic phenotype observed in KC may halt the fibrotic phenotype found in KC and promote expression and retention of the native stromal collagen ECM. Furthermore, targeting the metabolic shift to glycolysis observed in a number of pathological environments associated with ECM remodeling, including cancer [49,50] and lung fibrosis [51,52], may also prove as a novel therapeutic intervention to inhibit myofibroblast differentiation in the context of corneal scarring [53]. Clearly, further studies evaluating the effectiveness of antioxidants in reducing corneal thinning are required to determine drug efficacy in vivo.

The role of systemic factors in KC pathobiology has recently been proposed [17]. Consistent with gender dominance in terms of KC development, a number of studies have reported a higher male to female ratio within the KC population [35,54]. Previous studies have also identified a potential role for hormones in KC development [16,18]. In terms of KC severity, no significant differences have been found in men versus women with varying hormone statuses, yet increased corneal scarring has been associated with male KC patients compared to their female counterparts [55]. Consistent with this finding, evaluation of salivary hormone levels in KC patients showed little correlation with severity of the disease, though significant elevation in the androgen, dehydroepiandrosterone (DHEA) sulfate, and lower estrone levels in KC patients compared to healthy controls suggest that hormones may contribute to disease development [16]. The temporal and spatial regulation of DHEA has been found to be fundamentally important during neocortex formation in the developing embryo [56]. DHEA levels in humans appear to decrease significantly at birth followed by increased production by the adrenal gland around puberty [57], which corresponds to the average age of KC onset at around 15 years of age [17]. This correlative relationship does not necessarily equate to causality, though the role of hormones in postnatal physiology and their effects on cellular metabolism in the context of KC [58] remains a significant area of interest. Case studies have identified an increase in KC progression in certain patients following in vitro fertilization and hormone replacement [59,60]. Furthermore, altered expression of estrogen and androgen receptors [61] as well as thyroxine receptor [62] within the KC cornea has also been reported. Recent work has identified decreased systemic expression of a hormonally regulated factor, prolactin-inducible protein (PIP), in KC patients compared to healthy controls [17,18,63], which correlates with lower prolactin levels found in the KC aqueous humor [64]. Further work in the role of systemic hormones in influencing corneal structure is required to delineate the complex disease etiology involved in KC development and progression.

## 3. Effects of DM and KC on Corneal Structure

The two biomechanical properties that are important in defining the corneal structural and functional properties are elasticity and viscosity. The viscosity (e.g., stiffness) and elasticity (e.g., malleability) of the cornea is provided by organized collagen fibrils, associated proteoglycans, and fibrillin-containing microfibrils, whose distribution vary depending on the spatial location from the anterior to posterior stroma as well as from the central to peripheral cornea. ECM organization within the stroma allows the cornea to withstand internal and external stress due to intraocular pressure and environmental factors, respectively, while maintaining its characteristic dome shape, which is critical for proper visual acuity. The structural integrity of the cornea is mediated primarily via collagen and distributed proteoglycans that organize into stacked lamellae. Changes in collagen structure due to variations in the expression of the dominant collagen isoforms (e.g., type I, III, or V collagen) or proteoglycan distribution (e.g., keratocan, lumican, or decorin) may influence fibril diameter and organization within the cornea. Other microenvironmental factors, including high glucose levels or photooxidative CXL, may influence the number of crosslinks that form between collagen fibrils and thus affect the biomechanical properties of the tissue. All of the above alterations may lead to visual acuity problems.

### 3.1. Corneal Changes in DM

Diabetic complications affecting the cornea have been thoroughly reviewed [65,66,67,68]. DM has significant effects on the morphological, physiological, metabolic, and clinical aspects of the human cornea. The effects of DM manifest in multiple layers of the cornea: epithelium (e.g., keratopathy), peripheral nervous system (e.g., neuropathy), immune system (e.g., inflammation), stroma (e.g., collagen fibril changes), and endothelium (e.g., endothelial cell loss) [65]. DM often predisposes the ocular surface, including the cornea, to an increased risk of developing a number of conditions, such as persistent epithelial defects and dry eye [69], recurrent corneal erosions [70], and bacterial infection [71]. Moreover, an increase in morphological abnormalities, including altered nerve fiber density and neuropathic pain [72,73] and decreased corneal endothelial cell counts [74] are some of the other known ocular clinical manifestations of DM.

While systemic changes in metabolism occur in DM, cellular metabolism in corneal fibroblasts isolated from DM patients shows only modest changes in glucose metabolic pathways, e.g., glycolysis, tricarboxylic acid cycle, and the pentose phosphate pathway, with downregulation of some key glycolytic metabolites, including glyceraldehyde-3-phosphate and dihydroxyacetone phosphate [75]. Likewise, metabolic studies of corneal buttons isolated from DM patients showed altered tryptophan metabolism mediated via the kynurenine pathway, suggesting alternative pathways to glycolytic metabolism may also be involved in the pathological effects seen in the DM corneal stroma [75]. 

In terms of biological mechanisms, reduced growth factor expression and elevated proteinase activity may contribute to tissue degeneration over time. Studies have shown that DM leads to significant loss of corneal epithelial intermediate cells and basal epithelial cells [76]. Previous studies have also found lower hepatocyte growth factor (HGF) receptor c-met in diabetic corneas corresponding to reduced wound healing capacity [77]. Furthermore, increased matrix metalloproteinase (MMP)-3 and -10 expression in DM corneas suggests that the DM microenvironment within the stroma supports fibrotic ECM deposition and remodeling consistent with a wound healing phenotype [77,78]. Likewise, corneal fibroblasts isolated from DM patients express α-smooth muscle actin and secrete a thicker ECM high in type III collagen compared to healthy controls [75]. It is likely that the effects of DM on the subbasal nerve population may be a primary driver of the pathological effects associated with DM on the tissue. DM leads to morphological damage to the corneal sensory nerve population leading to reduced nerve fiber density and length [76]. 

The effects of DM on the cell populations found within the cornea also influence the structural and mechanical properties of the tissue. A study utilizing a rabbit model of alloxan-induced diabetes showed that elevated glucose for 8 weeks leads to significant increases in intraocular pressure, corneal thickness, and stiffness [79]. In human studies, DM resulted in a significant increase in corneal thickness by as much as 19% compared to non-DM controls with cases of neuropathy or elevated HbA_1C_ showing the highest change in thickness [80,81,82], though others have reported no significant difference in corneal thickness in DM [83,84]. Moreover, biomechanical changes in the cornea as a result of DM have shown conflicting results. In general, the corneal hysteresis and corneal resistance factor appeared to be significantly elevated in the DM population compared to controls, though various studies showed no effect or even decreased stiffness in DM patients (Table 1). The heterogeneity of the patient population, disease severity, the presence or absence of corneal edema, and the time of day of data collection may confound the biomechanical measurements set to determine the chronic effects of DM on the cornea, and thus likely contribute to significant variations from study to study.

In terms of collagen structure, DM has been associated with increased crosslinking mediated by AGEs that form following prolonged hyperglycemia. This process occurs in a parallel manner to the ‘browning’ phenomena reported in glucose-containing food items upon long-term storage or heating, which involves non-enzymatic glycation of proteins that adopt a yellowish-brownish color over time [90,91]. In terms of structural effects, AGE-mediated crosslinking in diabetic rats is associated with increased tendon stiffness [92] and higher mechanical strength that could be inhibited by insulin supplementation [93]. The presence of AGEs in the cornea has been shown to be localized to the laminin-rich epithelial basement membrane, which results in decreased epithelial cell attachment [94]. The altered biomechanics caused by AGE-mediated crosslinking have also been associated with decreased viscoelastic properties of the tissue with reduced fibril slippage, thereby increasing the brittle nature of the matrix and susceptibility to fracture or breakage of collagen fibrils. An in vitro study applying isolated rat tendons exposed to prolonged ribose showed increased collagen fibril density, thickness, and stiffness by one week [95]. During DM, AGE-mediated crosslinking also occurs on other abundant proteins with relatively long half-lives, including structural proteins, e.g., laminin, heparan sulfate proteoglycan, myelin, and tubulin, leading to defects in the basement membrane, endothelial barrier integrity, and maintenance of the macro- and microvasculature [30,96]. In general, corneas from DM patients may exhibit thickening of the basement membrane and reduced anchoring fibrils into the stroma that serve to bind the basement membrane to the stromal collagen [97]. Degeneration of the basement membrane in DM has also been reported [6].

### 3.2. Corneal Changes in KC

The normal progression of KC often causes biomechanical weakening of the cornea by altering the collagen arrangement and leading to ECM changes. KC has been associated with increased apoptosis of stromal keratocytes present in the anterior central cornea near Bowman’s layer in ~60% of KC corneas following transplantation based on a TUNEL assay compared to non-KC controls [98]. Pathological metabolic changes associated with increased oxidative stress in stromal keratocytes is thought to lead to altered collagen secretion favoring type III collagen over the dominant type I and V isoforms [39,99]. Defects in lysyl oxidase expression and/or activity in KC have also been reported [100,101,102]; however, lysyl oxidase mutations do not appear evident in all KC populations [103], highlighting that KC is a multifactorial disease with heterogeneous genetics. Changes in corneal nerves have been reported in KC, with reduced corneal sensitivity by as much as 21–26% detected in both KC and subclinical KC patients compared to controls [104,105]. Further cellular changes in the KC cornea that have been identified include increased MMP expression and activity (e.g., MMP-1, MMP-2, MMP-9) [106,107,108], elevated expression of pro-inflammatory mediators (e.g., tumor necrosis factor-α, interleukin-6) [109,110,111], and altered ECM-associated pathways (e.g., hydroxyproline, fibronectin, collagen) [112] in KC.

In terms of corneal structure, KC may present with up to a 60% decrease in central corneal thickness (<200 μm) for Stage 4 KC, based on the Amsler–Krumeich grading system [113]. Corneal thinning leads to detrimental effects on visual acuity and often corneal scarring due to severe steepening at the apex. Significant differences in the biomechanical parameters of early stages of KC (i.e., forme fruste KC) compared to controls may be difficult to identify in certain patients [114], though a number of studies have identified significantly reduced stiffness compared to controls based on the corneal hysteresis and corneal resistance factor [115,116] (Table 2). It is well-established that the advanced KC cornea shows significant differences in biomechanical properties, including reduced stiffness nearly 50% lower than controls [117,118,119]. The biomechanical changes observed in KC are a result of differential collagen fibril microstructure and organization within the central cornea. Immunohistochemical analysis of KC corneas has also shown alterations in basement membrane structure, as well as Bowman’s and Descemet’s layers [120] that may arise with severe curvature of the cornea that develops during later stages of the disease. Differences in collagen organization imaged by acoustic radiation force elastic microscopy have shown reduced branching and interweaving of collagen fibers in the anterior and posterior KC cornea compared to the normal basketweave pattern found in controls [118]. A number of studies have also identified differential angle directionality of collagen fibers in KC corneas, particularly at the corneal apex, which is the region most susceptible to thinning [121,122,123], further highlighting the defects in collagen organization that may contribute to the mechanical failings leading to ectasia. 

## 4. Epidemiological Studies Comparing DM and KC Prevalence

While DM is heavily associated with lifestyle (i.e., nutrition and activity level) that may contribute to prolonged hyperglycemia, correlations with similar systemic factors have not yet been attributed to KC development. A common similarity between the conditions is the potential role for oxidative stress in the pathological defects that may arise, including cell injury or death. Furthermore, systemic inflammation has also been identified in DM and KC, albeit with very different pro-inflammatory conditions. DM has been reported to present with subclinical systemic inflammation that affects several organs, including the pancreas, liver, and fat tissue, among others, which may contribute to organ dysfunction [126]. Several epidemiological studies have identified atopy and allergies as co-morbidities associated with the KC condition [127,128], though no such correlation appears evident in DM [129,130]. Clearly, differential systemic factors associated with each disease may have varying effects on the cellular response within the cornea that likewise modulates collagen expression and deposition.

Given the morphological and functional changes in the DM corneal stroma due to AGE-mediated crosslinking, the hypothesis that DM may be protective against KC development has been posited. Epidemiological studies comparing the prevalence of KC within the DM population have shown conflicting results (Table 3). Retrospective studies have identified an inverse association of KC development with DM, suggesting a protective effect of presumably AGE-mediated crosslinking on KC development or progression [131,132,133,134] with a single study showing a positive association of KC with T2DM development [135]. Woodward et al. identified 20% lower odds of KC development with DM that increased to 52% lower odds with severe DM [134], thus correlating the severity of DM, which is likely proportional to prolonged hyperglycemia or uncontrolled DM, to inhibition of corneal thinning. Whether this effect is related to AGE-mediated crosslinking or rather due to systemic differences that may blunt or inhibit KC development remains unknown. Of note, given the relatively young age of most KC patients (generally <40 years old), the protective role of T1DM, which is often associated with juvenile onset, may be more significant than T2DM with adult onset. The epidemiological studies thus far have focused largely on evaluating T2DM patient populations given the high prevalence within the general population.

## 5. Current Management Strategies for Corneal Defects in DM and KC

Treatment options for DM primarily target the systemic condition to control blood glucose levels and include lifestyle changes (e.g., diet and exercise), as well as pharmacological intervention (e.g., insulin, α-glucosidase inhibitors, and biguanides, among others). Treatments for corneal pathologies due to DM have likewise focused on lowering hyperglycemia within the tissue to reduce epithelial, stromal, and neuronal cell damage. While no systemic treatment has been proposed to specifically target corneal thinning in KC, the common treatments for KC, by contrast, focus on prosthetics to correct refractive error (e.g., contact lenses, scleral lenses, intrastromal ring segments), limit corneal thinning (e.g., CXL), or regain visual acuity loss to severe ectasia or scar development (e.g., penetrating keratoplasty (PKP)).

### 5.1. Management of DM

While treatments for the corneal defects that commonly present due to DM focus heavily on epithelial defects (e.g., keratopathy) and nerve degeneration (e.g., neuropathy), therapeutics targeting the stroma are, thus far, largely unexplored [65]. The primary treatment options for the DM cornea include targeting the systemic condition via normalizing blood glucose levels. Experimental therapeutics to treat corneal defects have been reported in DM animal models but are, thus far, not approved for clinical applications. These experimental treatments include topical insulin and small molecule therapeutics that target specific pathways associated with promoting wound healing (e.g., naltrexone) or cellular metabolism (e.g., aldose reductase inhibitors) and oxidative stress (e.g., antioxidants).

#### 5.1.1. Insulin

As the presence of chronic hyperglycemia in DM leads to significant disruption of cellular and tissue structure and function, therapeutics to restore glucose levels to baseline remains a practical approach to reduce DM pathology within the cornea. Insulin is a hormone normally released by the pancreas in response to elevated blood glucose levels that promotes cellular uptake of extracellular glucose. In situations of reduced insulin production (e.g., T1DM and possibly T2DM), administration of insulin may be performed to lower systemic glucose levels. Both systemic and topical application of insulin in DM subjects has shown improved wound healing of the skin [136,137]. In terms of ocular applications, similar positive therapeutic effects of insulin on the DM cornea have been posited. Protection against subbasal corneal nerve loss was found in a streptozocin (STZ)-induced DM rat model treated with topical insulin following 4 weeks post-DM induction independent of blood glucose levels [138]. Topical insulin applied to the cornea following epithelial debridement also appeared to promote re-epithelialization in an STZ-induced DM rat model with retention of corneal sensitivity similar to pre-injury controls [139]. Reduction in the epithelial defect in a small human clinical trial of 32 DM patients showed a similar favorable response to topical insulin treatment at 1-month post-operation, with a significant improvement in corneal epithelial recovery compared to controls treated with placebo [140]. These studies support the hypothesis that lowering extracellular glucose levels is essential to promoting proper corneal wound repair.

#### 5.1.2. Small-Molecule Therapeutics

Applications targeting the opioid receptor to improve DM wound healing using naltrexone have been reported. The mechanism of action appears to proceed via inhibition of the opioid growth factor receptor for promotion of fibroblast proliferation [141]. The topical application of naltrexone has shown high corneal biocompatibility and restoration of corneal sensitivity in a STZ-induced DM rat model [142]. Cocktail treatments of naltrexone and insulin did not appear to yield combinatorial effects when applied together, but both independently promoted DM wound healing [143]. 

Increased metabolic flux via the sorbitol pathway has previously been associated with DM complications [144]. Aldose reductase is involved in the first step of this process by mediating the conversion of glucose to sorbitol. In agreement with the sorbitol pathway playing a role in corneal defects associated with DM, inhibition of aldose reductase has shown positive effects on wound healing. Topical and oral administration of aldose reductase inhibitors (e.g., CT-112 and AL-1576, respectively) to galactose-fed rats showed retention of corneal sensitivity compared to untreated, galactose-fed controls at 7 months [145]. In a similar high-galactose canine model, oral administration of an aldose reductase inhibitor (e.g., epalrestat) following a high-glucose diet resulted in the protection of corneal epithelial barrier damage by 41 months post-diet commencement [146]. 

As DM is associated with increased inflammation, other approaches focusing on antioxidants have likewise been presented. The application of the potent natural product, curcumin, encapsulated within nanomicelles and administered intranasally to STZ-induced DM mice, showed improved re-epithelialization and corneal sensitization by 7 days post-debridement compared to untreated controls [147]. Additional growth factor/antioxidant cocktails have also been reported. For example, topical application of pigment epithelium-derived factor (PEDF) and docosahexaenoic acid (DHA) improved corneal re-epithelialization and nerve fiber retention and sensitivity in a STZ-induced mouse model 12 days post-epithelial debridement [148]. These results suggest that targeting the downstream effectors involved in DM may prove useful in preventing or delaying DM-associated pathologies.

### 5.2. Management of KC

KC is a progressive corneal disease that can lead to blindness (i.e., visual acuity ≤20/200) due to the inability to correct the refractive error as a result of an irregular astigmatism. A number of treatment options are available to improve visual acuity depending on the progression of the disease, which may proceed slowly or rapidly, as well as stabilize with time. The major goal of treatment is to improve visual acuity via prosthetics, e.g., spectacles, contacts, intrastromal ring segments, or via tissue replacement, e.g., PKP [15]. Recent advances in photooxidative CXL have developed as a novel approach to delay corneal thinning by stiffening the corneal stroma leading to reduced KC progression [149]. The prescription of spectacles or contact lenses may be suitable to correct vision in the early stages of KC. More severe cases involving the presence of a scar within the visual axis or extreme corneal curvature may require corneal transplantation. Below, we briefly discuss common management strategies available for the treatment of KC.

#### 5.2.1. Spectacles and Contact Lenses

With spectacles or glasses, achieving satisfactory visual acuity may be difficult depending on the corneal curvature and the presence of a severe astigmatism. The use of spectacles is often recommended for KC patients who can achieve 20/40 visual acuity or better. However, other options for correcting irregular astigmatism which cannot be met by spectacles include specialized contact lenses. For early stages of KC, the use of soft contact lenses may be applied to correct near-sightedness and astigmatism. In cases where the disease has progressed to a later stage, rigid gas-permeable lenses or several other specialized lenses, such as hybrid lenses, piggyback lenses, or scleral lenses, are recommended. Other specialized ocular prosthetics include rigid gas-permeable, hybrid, and piggyback lenses, which involve the inclusion of multiple lenses to correct the refractive error. Scleral lenses, which fit directly to the scleral tissue rather than the irregular corneal surface, are often prescribed for advanced KC stages to reduce physical irritation induced by an ill-fitting lens. 

#### 5.2.2. Penetrating Keratoplasty 

Penetrating keratoplasty (PKP) is a common procedure for rectifying loss in visual acuity in KC that may occur in late stages of the disease. In general, about 10–20% of KC patients progress to a severity requiring PKP to recover the loss in visual acuity [15]. Corneal transplantation is the only option recommended for improving visual acuity in such conditions as even the wearing of contact lenses does not help in improving visual outcome due to the presence of a severe ectatic corneal surface. Studies have shown high success rates of 87–98% for PKP in KC patients [150]. PKP remains a successful procedure for the treatment of late stages of KC to recover functional vision. Visual correction following PKP may be required with studies showing up to 30–47% of KC patients undergoing PKP require either spectacles or contacts, respectively, by 18 months post-operation [150]. Patient recovery following PKP usually takes 4–6 weeks, though up to 1–2 years for stabilization of visual acuity [151]. Some of the complications related to PKP include donor rejection, posterior subcapsular cataract, and glaucoma, among others, though development of these complications following PKP is generally considered rare [152]. Other limitations include the possible need for a second transplant due to recurrent KC development in the first graft [153,154], though also relatively uncommon. Availability to adequate pre- and post-operative healthcare, as well as the limitations in donor tissue availability, remain significant challenges in the treatment of corneal dystrophies requiring transplantation. 

#### 5.2.3. Corneal Crosslinking 

CXL was introduced in 1997 as a non-invasive procedure to increase corneal stiffness in the context of KC [155,156]. In 2016, CXL was approved by the US Food and Drug Administration and, ever since then, has been extensively used in the treatment of KC and secondary corneal ectasia at varying stages of disease progression. According to a recent study report, there has been a significant reduction in annual keratoplasties by up to 25% performed in the 3 years since the introduction of CXL treatment [157]. The reduction in PKPs is likely a result of a high success rate of KC stabilization characterized by reduced corneal curvature and improvement in visual acuity. 

CXL is a relatively easy, well-documented procedure involving riboflavin and UV-A exposure. Riboflavin serves as a photosensitizer to enable free-radical formation within the stromal ECM, thus allowing the formation of covalent bonds within and between collagen fibrils. The presence of riboflavin may also further restrict the damage to the lens or retina due to UV-A radiation exposure. The Dresden protocol is the established method for CXL and involves removal of the corneal epithelium and pre-incubation of the cornea with a riboflavin solution followed by UV-A irradiation [158]: (1) 0.1% (w/v) riboflavin-5-phosphate in a dextran solution is applied to the debrided cornea 30 minutes prior to UV-irradiation and at 5 minute intervals during UV-exposure; (2) UV-A light exposure (λ_370nm_ = 30 min) induces crosslink formation. Alternative methods, including accelerated CXL with shortened exposure time and higher irradiance, have been proposed [159]. 

The risk for developing secondary complications of the procedure, such as recurrent epithelial defects, infectious keratitis, and dry eye, is present, and is likely a result of post-operative contact lens use. Another potential limitation of CXL is that it is not suitable for all individuals with KC, including those with history of infection (e.g., herpes simplex) or recurrent erosions, as well as those with severe KC or corneal scarring. However, various modifications have been made to the conventional CXL method so far to avoid possible complications associated with the CXL treatment. Overall, CXL is a promising technique for arresting KC progression and additional innovation of this approach will likely improve the treatment efficacy and clinical outcome.

## 6. Chemistry of Collagen Crosslinking

Applications of chemical crosslinking are widely used in biomedical research to mediate stable fixation of tissues, including formaldehyde and glutaraldehyde, which mediate crosslinking of proteins by converting the primary amine of lysine to a reactive iminium ion, which then condenses with adjacent tyrosine molecules to form a covalent crosslink [160]. Depending on the tissue, these reagents exhibit cellular toxicity and carcinogenic properties [161] and are thus not compatible with most in vivo applications. A number of nontoxic chemical crosslinkers have been reported in recent years, including disuccinimidyl glutarate (DSG), which relies on NHS-ester chemistry mediated primarily by activation of lysine groups [162]. Other crosslinkers, such as diazirine photoreactive agents, apply long-wave UV light (330–370 nm) excitation to generate an activated intermediate that can crosslink nearby groups. In this section, we discuss three major reactions of collagen crosslinking important in corneal biology: (1) lysyl oxidase-mediated crosslinking as a post-translational modification of secreted collagen that stabilizes collagen fibril structure; (2) advanced glycation end product (AGE)-mediated crosslinking that occurs as a result of reaction of glycated proteins, including collagen, to non-enzymatically react with surrounding proteins; and (3) photooxidative CXL mediated by riboflavin as a treatment option for KC and corneal ectasia. 

### 6.1. Lysyl Oxidase-Mediated Crosslinking 

**Figure 3:**
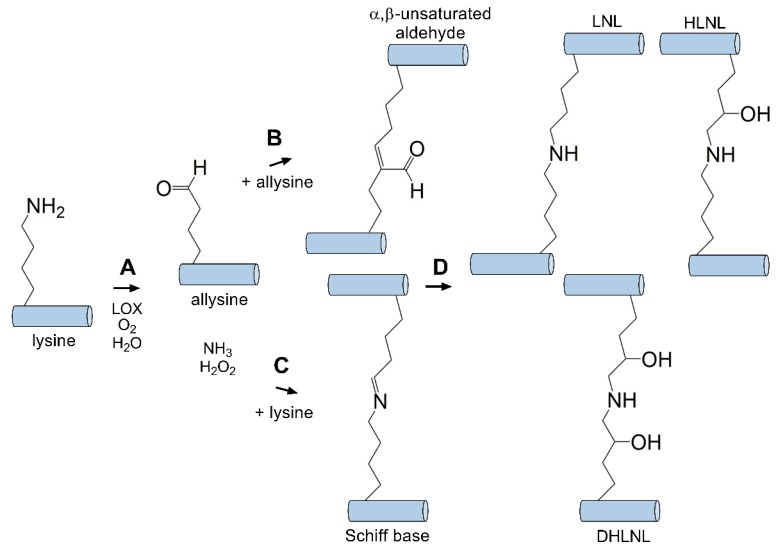
Lysyl oxidase (LOX) mediates intermolecular crosslinks between tropocollagen molecules. (**A**) LOX catalyzes the conversion of a primary amine to an aldehyde (allysine). This intermediate can then react with an adjacent (**B**) aldhyde (allysine) or (**C**) primary amine (lysine) to generate an α,β-unsaturated aldehyde or Schiff base intermediate, respectively. (**D**) Condensation generates stable covalently bound crosslinks (e.g., lysinonorleucine (LNL), dihydroxylysinorleucine (DHLNL), hydroxylysinonorleucine (HLNL) [166,167]) between tropocollagen molecules.

Physiological crosslinking occurs in nearly every tissue in the body containing collagen, from bone [163] to the skin [164] and cornea [101]. This enzymatic reaction is required for the formation of a stable ECM structure to support cellular attachment and proper rigidity. Lysyl oxidase is a copper amine oxidase primarily responsible for catalyzing the formation of crosslinks between collagen fibrils by generating reactive aldehydes on lysine residues found in the telopeptide domain of collagen that may cross-react with nearby groups [165]. This reaction proceeds with conversion of an ε-amine (e.g., lysine) group to an aldehyde (e.g., allysine) catalyzed by lysyl oxidase (Figure 3). This aldehyde group may then react with other surrounding ε-amine moieties or another proximal reactive aldehyde to generate a Schiff base or α,β-unsaturated aldehyde, respectively. Spontaneous condensation of these species occurs to generate crosslinks between collagen molecules consisting of lysinorleucine, dihydroxylysinorleucine, hydroxylysinonorleucine, and histidinohydroxylysinonorleucine, which have all been detected within the cornea [166,167]. Lysyl oxidase is secreted as an inactive proenzyme that is cleaved by procollagen C-proteinase to an active form. A number of growth factors, including transforming growth factor-β1 (TGF-β1) and platelet-derived growth factor (PDGF), are known to increase the expression of lysyl oxidase, thus resulting in elevated collagen crosslinking during conditions associated with elevated levels, including wound healing [165] and cancer metastasis [168]. Stiffening of the ECM due to enhanced lysyl oxidase activity is also associated with cancer progression and fibrosis [169,170]. Downregulation of lysyl oxidase is known to be promoted by basic fibroblast growth factor (bFGF) in osteoblasts. Furthermore, conditions associated with defects in lysyl oxidase-mediated crosslinking, such as Ehlers–Danlos syndrome [171] and Menkes syndrome [172] lead to congenital heart conditions and severe bone and skin defects similar to features observed with copper-deficiency. By contrast, conditions associated with hypertrophic scar have also been associated with increased lysyl oxidase activity, suggesting that strict regulation of this enzyme is important to minimize fibrosis as well as maintain proper ECM structure [173,174].

### 6.2. Advanced Glycation End Product-Mediated Crosslinking

**Figure 4:**
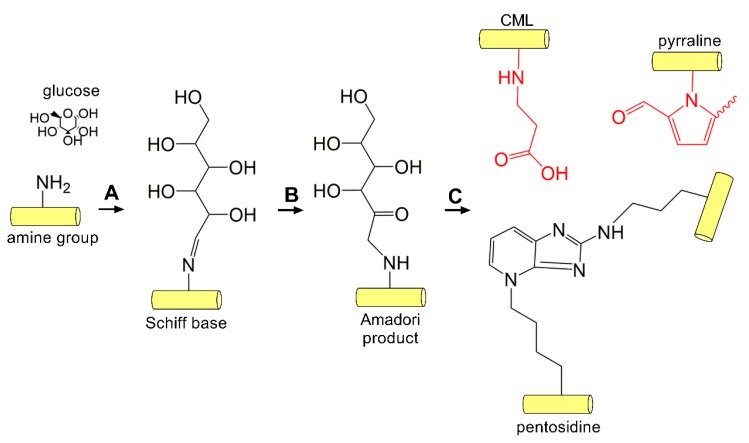
AGE-mediated crosslinking. (**A**) Glucose-mediated crosslinking begins via the Maillard reaction involving the addition of an aldehyde sugar to the amine group found on lysine or arginine side chains generating a Schiff base followed by (**B**) rearrangement to the Amadori product. (**C**) AGE adducts (red, N6-carboxymethyllysine (CML) and pyrraline) and inter- and intramolecular crosslinks (pentosidine) form on collagen within the cornea [178,179].

Compared to enzymatic crosslinking by lysyl oxidase, non-enzymatic AGE-mediated crosslinking can occur between collagen helices between lysine and histidine or arginine, and thus contribute to stable crosslinks that are resistant to enzymatic degradation [175]. Non-enzymatic crosslinking in DM occurs primarily via the Maillard reaction. The Maillard reaction was first reported in 1912 by Louis-Camille Maillard [176], and is associated with the non-enzymatic reaction of proteins and sugars giving rise to a brown color change and release of carbon dioxide. Formation of these glycated proteins leads to changes in color, taste, and aroma, thus playing a significant role in food and agriculture. The classical reaction scheme involves the glycation of hemoglobin by the reaction of the aldehyde group of glucose with the β-chain of hemoglobin. This reaction gives rise to the glycated HbA_1C_, an established serum biomarker of sustained elevated glucose levels [177]. The Maillard reaction proceeds via conversion of a primary amine found on amino acid residues, such as lysine or arginine, that are then converted to a reactive Schiff base, which rearranges to form an Amadori product (Figure 4). Studies have identified increased end products of the Maillard reaction, including pentosidine, within the diabetic cornea, likely produced as a result of lysine glycation followed by reaction with arginine during prolonged hyperglycemia [178,179].

### 6.3. Photooxidative Crosslinking Mediated by Riboflavin 

**Figure 5:**
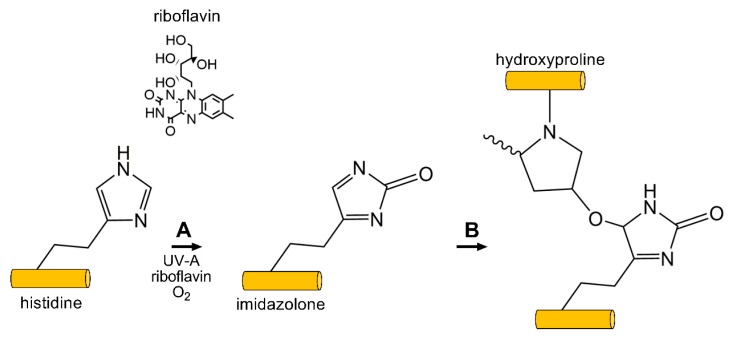
Photooxidative CXL commonly uses the photoxidizing agent riboflavin, and exposure to short wavelength UV light (360 nm) to generate free-radicals on ECM proteins, e.g., collagen and proteoglycans. (**A**) Singlet-oxygen reacts with histidine (shown) or other reactive carbonyl groups forming an imidazolone intermediate. (**B**) A reactive nucleophile, such as the hydroxyl group on hydroxyproline (shown), threonine, or tyrosine, reacts with the imidazolone intermediate generating inter- and intramolecular crosslinks between and within collagen and surrounding proteoglycans.

In contrast to LOX- and AGE-mediated crosslinking, the amine group present on lysine or arginine are thought to not be directly involved in crosslink formation in photooxidative CXL with riboflavin [180]. The carbonyl groups found on histidine, hydroxyproline, tyrosine, or threonine appear to be the dominant functional group, as masking of the carboxyl moiety via chemical blockage using hydroxylamine or sodium azide inhibits crosslink formation [180]. This mechanism proceeds via singlet oxygen formation, thus relying on the carbonyl group present on the side chain of select amino acids following exposure to UV light (360 nm) in the presence of riboflavin [181] (Figure 5). Riboflavin functions as a photosensitizer in the presence of UV light, thereby absorbing light leading to conversion to an excited singlet riboflavin molecule that rapidly transforms to a triplet state [181]. This reactive intermediate can then mediate hydride or electron transfer to collagen or other ECM proteins, which then converts to covalent bonds between substrates in a Type 1 process. Photooxidative CXL with riboflavin can also proceed via reaction of activated riboflavin with oxygen generating ROS, which can then mediate CXL between substrates in a Type II process. The primary amino acids thought to be involved in this reaction are hydroxyproline, hydroxylysine, tyrosine, or threonine, forming intramolecular and intermolecular bonds within the collagen backbone and with surrounding proteoglycans [181]. Photooxidative CXL with riboflavin is also expected to proceed, via AGE-mediated mechanisms, primarily between highly glycosylated proteoglycans found in the stroma, e.g., keratocan and lumican, as well as collagen fibrils [182]. The formation of dityrosine crosslinks have also been reported and may play a further role in tissue stiffening post-CXL [183].

## 7. Effects of Crosslinking on Corneal Structure

To target corneal thinning in KC, CXL is a technique that has garnered a lot of interest in the field of vision and ocular disease. CXL is considered an exogenous, minimally invasive treatment that may stunt disease progression by stiffening the collagen bonds found within the stroma. A number of studies have successfully evaluated treatment efficacy and the short- and long-term safety profile of CXL in halting the progression of corneal thinning in KC and secondary corneal ectasia [184]. In this section, we detail some of the biomechanical changes of the cornea witnessed post-CXL treatment, and provide critical information about the changes observed at the ultrastructural and microenvironment level, including cellular responses and ROS generation. 

### 7.1. Biomechanical Changes of the Cornea Post-Crosslinking

CXL treatment is the only approved approach, to date, for progressive KC and corneal ectasia that can delay or block disease progression and further reduce the demand for donor keratoplasty. Crosslinking as a result of lysyl oxidase activity, AGE reactivity, or riboflavin/UV-A exposure leads to biomechanical changes in ECM structure. While AGE-mediated crosslinking and photooxidative CXL occur via chemical mechanisms that favor covalent bonding by reactive lysine/arginine groups versus reactive carbonyl groups, respectively, both approaches appear to significantly increase corneal stiffness. Crosslinked collagen has been shown to be more resistant to proteolytic degradation [185]. Furthermore, the effects of CXL on corneal stiffness may contribute to elevated intraocular pressure readings obtained using Goldmann applanation tonometry [186], likely a result of increased tissue rigidity due to crosslink formation [187]. 

**Figure 6:**
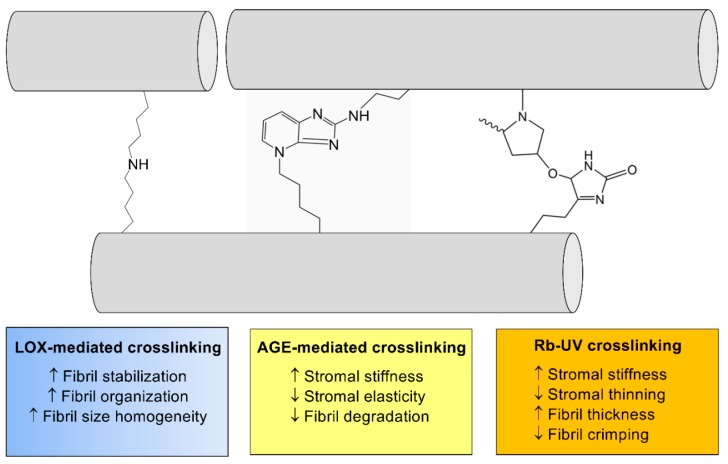
The general effects of crosslinking on collagen structure within the corneal stroma. Enzymatic crosslinking (lysyl oxidase (LOX)-mediated (blue)) and non-enzymatic crosslinking (AGE-mediated (yellow) and riboflavin (Rb)/UV-mediated (orange)) lead to significant effects on ECM structure via modifications to collagen fibril (gray) thickness and organization.

The structural modifications of the cornea due to CXL are thought to primarily occur within the collagenous-rich stroma (Figure 6). Ex vivo studies on the effects of CXL on human and porcine corneal tissue samples have demonstrated its association with increased collagen fiber diameter by ~12% in the anterior cornea exposed to the highest direct UV-A irradiation and ~5% in the posterior cornea [188]. Some notable study findings include the study conducted by Wollensak et al., where the rigidity of the cornea was observed to have increased by 328.9% after collagen crosslinking [189] and increased Young’s modulus up to 8 months post-CXL with no significant difference in corneal thickness [190]. The effects of CXL on collagen structure have shown decreased collagen waviness or crimping in the anterior corneal stroma by 1-month post-riboflavin-mediated CXL in a rabbit model [191]. Riboflavin-mediated CXL of the cornea has also been associated with loss of corneal transparency by 1 month, though resolution of the haze is evident by 3 months post-CXL in rabbit models [191]. In a human clinical trial, the efficacy of CXL determined based on the maximum keratometry in the crosslinked group compared to sham controls showed an improvement in corneal steepening (K_max_) by 1.6 diopters with a parallel improvement in visual acuity by at least 1 line (corrected distance visual acuity) at 1-year post-CXL [192]. Other studies have also concluded that CXL can help to reduce progression due to secondary corneal ectasia post-refractive surgery with similar halting of corneal steepening and stabilization of visual acuity [193].

### 7.2. Cellular Changes in the Cornea Post-Crosslinking

A number of studies have evaluated the effects of crosslinking on the cell populations found within the cornea. In terms of AGE-mediated crosslinking, crosslinked adducts can bind to the receptor for advanced glycation end products (RAGEs), which are pro-inflammatory receptors expressed on lymphocytes among other cell types that recognize various ligands [194]. Their binding to AGEs may be particularly important in promoting inflammatory processes associated with DM.

In terms of photooxidative crosslinking, riboflavin/UV-A exposure has been reported to show cytotoxicity towards keratocytes in vitro at a much lower irradiance (0.5 mV/cm^2^) compared to UV-A exposure alone [195], suggesting that riboflavin-mediated ROS production may be key to this observed cytotoxicity. Studies have likewise shown a reduction in the keratocyte population found in the anterior cornea by 1 to 6 months post-CXL that appears to recover by 12 months to pre-operative levels, as assessed by in vivo confocal microscopy [196,197]. The CXL reaction may lead to cellular damage through the production of ROS, which may be cytotoxic at high concentrations to other cell types within the cornea, including the endothelium [198]. 

In terms of downstream effectors modulated by CXL, in vitro studies evaluating the effects of CXL on primary corneal stromal fibroblasts isolated from KC patients have shown that CXL downregulates canonical TGF-β signaling based on lower pSMAD2/3 expression post-CXL in KC [199]. A normalization of lysyl oxidase abundance [199], increased proteoglycan expression (e.g., lumican, mimecan, and decorin) [200], and a reduction in lactate levels in KC-derived corneal fibroblasts following CXL have been reported [200] suggesting that riboflavin/UV-A CXL may modulate ECM deposition and collagen crosslinking at the cellular level in addition to the direct structural modifications to collagen. Given a prominent role for oxidative stress in corneal dystrophies including KC [201], further studies are warranted to distinguish the potent antioxidant properties of riboflavin [202] on the KC phenotype independent of UV-A irradiation.

## 8. Potential Role for AGE-Mediated Crosslinking in KC Prevention

Given the clear therapeutic effectiveness of riboflavin-mediated CXL in the treatment of KC, further studies evaluating the role of other crosslinking reactions in stromal thickening are warranted. DM provides a microenvironment conducive to AGE-mediated crosslinking due to the often chronic exposure of peripheral tissues to elevated glucose. Though the epidemiological studies remain inconclusive regarding a protective effect of DM against KC development, the biomechanical changes that occur in the DM cornea are relatively consistent in showing an increase in corneal thickness with no to moderate stiffening, which appears to vary heavily between studies (Table 1). One possible cause for the paucity of clinical evidence for protection is the limited patient population size with juvenile-onset T1DM, compared to adult-onset T2DM, and the relatively rare occurrence of KC. Of particular interest is the question that remains as to whether AGE-associated adducts in the cornea promote pathological effects mediated via RAGE-activation or whether AGE crosslinks are innocuous yet lead to stiffening of the corneal stroma that may limit KC progression.

In addition to DM, crosslinking also occurs during aging, with formation of crosslinked adducts on long-lived proteins, such as collagen and elastin. This process has been heavily studied in the human ocular lens, which is a non-regenerative tissue and, thus, many of the lens proteins remain within the host from birth to death [203] in the absence of surgical removal and replacement, such as cataract surgery. Studies have suggested that with aging, protein aggregation and crosslinking may occur via oxidation, racemization, and other post-translational modifications of lens proteins [204]. While KC is known to stabilize with age (generally by the third to fourth decade of life), it remains unclear if crosslinking as a result of aging halts KC progression. However, a direct role of aging in KC stabilization seems unlikely as these slow reactions may not lead to functional tissue changes until much later in life (sixth to seventh decade of life).

## 9. Summary and Conclusions

Enzymatic crosslinking (e.g., lysyl oxidase-mediated) and non-enzymatic crosslinking (e.g., AGE- and riboflavin/UV-A-mediated) are important in the context of tissue health and disease. DM is associated with increased AGEs that lead to inter- and intramolecular crosslinking and formation of AGE adducts on long-lived proteins, such as collagen and elastin. AGE species are thought to also form as a result of aging. This process results in ECM stiffening in various tissues, including the cornea. Whether this crosslinking phenomenon results in modest protection from KC development remains inconclusive. Given the practical effectiveness of photooxidative crosslinking in the treatment of KC, induction of crosslinks between collagen fibrils leads to stabilization of corneal thinning, likely as a result of increased rigidity of the tissue and decreased susceptibility to degradation. Furthermore, the effects of photooxidative crosslinking on corneal fibroblasts derived from KC patients appear to promote more favorable cellular metabolism. Additional studies are warranted to determine if reactivity of AGEs may promote further stabilization of KC.

## Figures and Tables

**Table 1:** Effects of DM on corneal thickness and biomechanical parameters in human patients.

Study	System	Sample Size	Corneal Thickness	Biomechanical Parameters	Ref.
Goldich et al., 2009	DM patients and controls	n = 40 (DM); n = 40 (Control)	548 ± 33 μm (DM); 530 ± 36 μm (Control)	↑ Corneal hysteresis and resistance factor	[85]
Hager et al., 2009	DM patients and controls	n = 99 (DM); n = 385 (Control)	554 ± 50 μm (DM); 542 ± 40 μm (Control)	↑ Corneal hysteresis with no significant difference in corneal thickness	[83]
Sahin et al., 2009	DM patients and controls	n = 81 (DM); n = 120 (Control)	550 ± 41 μm (DM); 535 ± 39 μm (Control)	↓ Corneal hysteresis with no significant difference in corneal resistance factor	[86]
Kotecha et al., 2010	DM patients and controls	n = 61 (DM); n = 123 (Control)	550 ± 38 μm (DM); 550 ± 33 μm (Control)	↑ Corneal resistance factor with no significant difference in corneal thickness or corneal hysteresis	[84]
Pérez-Rico et al., 2015	DM patients and controls	n = 94 (DM); n = 41 (Control)	561–565 (± 35–38) μm (DM); 516 ± 34 μm (Control)	↓ Corneal hysteresis and ↓ deformation amplitude in DM correlating with HbA_1C_ levels	[87]
Beato et al., 2019	DM patients and controls	n = 60 (DM); n = 48 (Control)	558 ± 35 μm (DM); 558 ± 30 μm (Control)	No significant difference in corneal hysteresis and resistance factor	[88]
Ramm et al., 2019	DM patients and controls	n = 35 (DM); n = 35 (Control)	556 ± 32 μm (DM); 548 ± 33 μm (Control)	↑ Corneal hysteresis and resistance factor	[89]

**Table 2:** Effects of KC and forme fruste KC on corneal thickness and biomechanical parameters in human patients.

Study	System	Sample Size	Corneal Thickness	Biomechanical Parameters	Ref.
Ahmadi Hosseini et al., 2014	Mild to moderate KC patients and myopic controls	n = 50 (KC); n = 50 (Control)	492 ± 30 μm (KC); 547 ± 33 μm (Control)	↓ Corneal hysteresis and ↓ corneal resistance factor; differences between central and peripheral regions	[115]
Tian et al., 2014	KC patients and controls	n = 52 (KC); n = 52 (Control)	472 ± 50 μm (KC); 530 ± 26 μm (Control)	↑ Deformation amplitude, ↑ curvature	[124]
Ayar et al., 2015	KC and forme fruste KC patients and controls	n = 27 (KC); n = 40 (Control)	481 ± 31 μm (KC); 496 ± 35 μm (KC); 548 ± 29 μm (Control)	↓ Corneal hysteresis and ↓ corneal resistance factor	[116]
Luz et al., 2016	Forme fruste KC patients and controls	n = 21 (KC); n = 76 (Control)	527 ± 17 μm (KC); 530 ± 26 μm (Control)	No significant difference in corneal hysteresis or corneal resistance factor; Significant difference based on tomographic parameters	[114]
Pẽna-Garcia et al., 2016	Subclinical KC patients and controls	n = 28 (KC); n = 184 (Control)	512 ± 31 μm (KC); 542 ± 30 μm (Control)	Significant differences in max deformation amplitude and first applanation time	[125]

**Table 3:** Summary of epidemiological studies comparing DM and KC prevalence in human populations.

Study	Study Size (*n*)	Design	Findings	Association	Ref.
Seiler et al., 2000	KC patients (n = 571) non-KC controls (n = 571)	Retrospective case-control study	T2DM showed a protective effect against KC development (odds ratio = 0.2195)	Inverse association of KC development with DM	[131]
Kuo et al., 2006	KC patients without DM (n = 269) KC patients with DM (n = 26)	Retrospective cross-sectional study	T2DM showed a protective effect against more severe KC (odds ratio = 0.2); No difference in DM prevalence in KC population	Inverse association of DM with KC severity	[132]
Kosker et al., 2014	KC patients (n = 1377) non-KC controls (n = 4131) and T2DM KC patients (n = 75) non-DM KC controls (n = 225)	Retrospective case-control and Cross-sectional study	Higher prevalence of T2DM in KC population compared to controls (6.75% and 4.84%, respectively); Higher severity of KC in DM patients (odds ratio = 2.691)	Positive association of KC development with T2DM	[135]
Naderan et al., 2014	KC patients (n = 1383) non-KC controls (n = 1383)	Retrospective case-control study	T2DM showed a protective effect against KC development (odds ratio = 0.350)	Inverse association of KC development with DM	[133]
Woodward et al., 2016	KC patients (n = 16,053) non-KC controls (n = 16,053)	Retrospective longitudinal cohort study	20% lower odds of KC development with uncomplicated DM; 52% lower odds of KC development with DM-associated organ failure	Inverse association of KC development with DM	[134]

## References

[1] Shoulders M.D., Raines R.T. (2009). Collagen structure and stability. Annu. Rev. Biochem..

[2] Birk D.E., Fitch J.M., Babiarz J.P., Doane K.J., Linsenmayer T.F. (1990). Collagen fibrillogenesis in vitro: Interaction of types I and V collagen regulates fibril diameter. J. Cell Sci..

[3] Marchant J.K., Zhang G., Birk D.E. (2002). Association of type XII collagen with regions of increased stability and keratocyte density in the cornea. Exp. Eye Res..

[4] Meek K.M., Knupp C. (2015). Corneal structure and transparency. Prog. Retin. Eye Res..

[5] Kapoor R., Sakai L.Y., Funk S., Roux E., Bornstein P., Sage E.H. (1988). Type VIII collagen has a restricted distribution in specialized extracellular matrices. J. Cell Biol..

[6] Ljubimov A.V., Burgeson R.E., Butkowski R.J., Michael A.F., Sun T.-T., Kenney M.C. (1995). Human corneal basement membrane heterogeneity: Topographical differences in the expression of type IV collagen and laminin isoforms. Lab. Investig..

[7] Chen S., Mienaltowski M.J., Birk D.E. (2015). Regulation of corneal stroma extracellular matrix assembly. Exp. Eye Res..

[8] Kadler K.E., Holmes D.F., Trotter J.A., Chapman J.A. (1996). Collagen fibril formation. Biochem. J..

[9] Linsenmayer T.F., Gibney E., Igoe F., Gordon M.K., Fitch J.M., Fessler L.I., Birk D.E. (1993). Type V collagen: Molecular structure and fibrillar organization of the chicken alpha 1(V) NH2-terminal domain, a putative regulator of corneal fibrillogenesis. J. Cell Biol..

[10] Holmes D.F., Kadler K.E. (2005). The precision of lateral size control in the assembly of corneal collagen fibrils. J. Mol. Biol..

[11] Chen S., Birk D.E. (2013). The regulatory roles of small leucine-rich proteoglycans in extracellular matrix assembly. FEBS J..

[12] Tao Z., Shi A., Zhao J. (2015). Epidemiological Perspectives of Diabetes. Cell Biochem. Biophys..

[13] Charkaluk M.L., Czernichow P., Levy-Marchal C. (2002). Incidence data of childhood-onset type I diabetes in France during 1988-1997: The case for a shift toward younger age at onset. Pediatr. Res..

[14] Schoenle E.J., Lang-Muritano M., Gschwend S., Laimbacher J., Mullis P.E., Torresani T., Biason-Lauber A., Molinari L. (2001). Epidemiology of type I diabetes mellitus in Switzerland: Steep rise in incidence in under 5 year old children in the past decade. Diabetologia.

[15] Rabinowitz Y.S. (1998). Keratoconus. Surv. Ophthalmol..

[16] McKay T.B., Hjortdal J., Sejersen H., Asara J.M., Wu J., Karamichos D. (2016). Endocrine and Metabolic Pathways Linked to Keratoconus: Implications for the Role of Hormones in the Stromal Microenvironment. Sci. Rep..

[17] Sharif R., Bak-Nielsen S., Hjortdal J., Karamichos D. (2018). Pathogenesis of Keratoconus: The intriguing therapeutic potential of Prolactin-inducible protein. Prog. Retin. Eye Res..

[18] Sharif R., Bak-Nielsen S., Sejersen H., Ding K., Hjortdal J., Karamichos D. (2019). Prolactin-Induced Protein is a novel biomarker for Keratoconus. Exp. Eye Res..

[19] Shaw J.E., Sicree R.A., Zimmet P.Z. (2010). Global estimates of the prevalence of diabetes for 2010 and 2030. Diabetes Res. Clin. Pract..

[20] Association A.D. (2018). 2. Classification and Diagnosis of Diabetes: Standards of Medical Care in Diabetes-2018. Diabetes Care.

[21] Herman W.H., Ma Y., Uwaifo G., Haffner S., Kahn S.E., Horton E.S., Lachin J.M., Montez M.G., Brenneman T., Barrett-Connor E. (2007). Differences in A1C by race and ethnicity among patients with impaired glucose tolerance in the Diabetes Prevention Program. Diabetes Care.

[22] Spanakis E.K., Golden S.H. (2013). Race/ethnic difference in diabetes and diabetic complications. Curr. Diabetes Rep..

[23] Kautzky-Willer A., Harreiter J., Pacini G. (2016). Sex and Gender Differences in Risk, Pathophysiology and Complications of Type 2 Diabetes Mellitus. Endocr. Rev..

[24] Baekkeskov S., Aanstoot H.J., Christgau S., Reetz A., Solimena M., Cascalho M., Folli F., Richter-Olesen H., De Camilli P. (1990). Identification of the 64K autoantigen in insulin-dependent diabetes as the GABA-synthesizing enzyme glutamic acid decarboxylase. Nature.

[25] Baekkeskov S., Nielsen J.H., Marner B., Bilde T., Ludvigsson J., Lernmark A. (1982). Autoantibodies in newly diagnosed diabetic children immunoprecipitate human pancreatic islet cell proteins. Nature.

[26] Lan M.S., Wasserfall C., Maclaren N.K., Notkins A.L. (1996). IA-2, a transmembrane protein of the protein tyrosine phosphatase family, is a major autoantigen in insulin-dependent diabetes mellitus. Proc. Natl. Acad. Sci. USA.

[27] Boelaert K., Newby P.R., Simmonds M.J., Holder R.L., Carr-Smith J.D., Heward J.M., Manji N., Allahabadia A., Armitage M., Chatterjee K.V. (2010). Prevalence and Relative Risk of Other Autoimmune Diseases in Subjects with Autoimmune Thyroid Disease. Am. J. Med..

[28] Smyth D.J., Plagnol V., Walker N.M., Cooper J.D., Downes K., Yang J.H.M., Howson J.M.M., Stevens H., McManus R., Wijmenga C. (2008). Shared and Distinct Genetic Variants in Type 1 Diabetes and Celiac Disease. N. Engl. J. Med..

[29] Nolan C.J., Damm P., Prentki M. (2011). Type 2 diabetes across generations: From pathophysiology to prevention and management. Lancet.

[30] Brownlee M. (1992). Glycation products and the pathogenesis of diabetic complications. Diabetes Care.

[31] Testa R., Bonfigli A.R., Prattichizzo F., La Sala L., De Nigris V., Ceriello A. (2017). The “metabolic memory” theory and the early treatment of hyperglycemia in prevention of diabetic complications. Nutrients.

[32] Zhang L., Chen B., Tang L. (2012). Metabolic memory: Mechanisms and implications for diabetic retinopathy. Diabetes Res. Clin. Pract..

[33] Basile K.J., Johnson M.E., Xia Q., Grant S.F. (2014). Genetic susceptibility to type 2 diabetes and obesity: Follow-up of findings from genome-wide association studies. J. Endocrinol..

[34] Rosen E.D., Kaestner K.H., Natarajan R., Patti M.E., Sallari R., Sander M., Susztak K. (2018). Epigenetics and Epigenomics: Implications for Diabetes and Obesity. Diabetes.

[35] Godefrooij D.A., de Wit G.A., Uiterwaal C.S., Imhof S.M., Wisse R.P. (2017). Age-specific Incidence and Prevalence of Keratoconus: A Nationwide Registration Study. Am. J. Ophthalmol..

[36] Kennedy R.H., Bourne W.M., Dyer J.A. (1986). A 48-year clinical and epidemiologic study of keratoconus. Am. J. Ophthalmol..

[37] Olivares Jimenez J.L., Guerrero Jurado J.C., Bermudez Rodriguez F.J., Serrano Laborda D. (1997). Keratoconus: Age of onset and natural history. Optom. Vis. Sci..

[38] Kenney M.C., Brown D.J., Rajeev B. (2000). Everett Kinsey lecture. The elusive causes of keratoconus: A working hypothesis. CLAO J..

[39] Karamichos D., Hutcheon A.E., Rich C.B., Trinkaus-Randall V., Asara J.M., Zieske J.D. (2014). In vitro model suggests oxidative stress involved in keratoconus disease. Sci. Rep..

[40] Karamichos D., Zieske J.D., Sejersen H., Sarker-Nag A., Asara J.M., Hjortdal J. (2015). Tear metabolite changes in keratoconus. Exp. Eye Res..

[41] Chwa M., Atilano S.R., Hertzog D., Zheng H., Langberg J., Kim D.W., Kenney M.C. (2008). Hypersensitive response to oxidative stress in keratoconus corneal fibroblasts. Investig. Ophthalmol. Vis. Sci..

[42] McKay T.B., Hjortdal J., Priyadarsini S., Karamichos D. (2017). Acute hypoxia influences collagen and matrix metalloproteinase expression by human keratoconus cells in vitro. PLoS ONE.

[43] Atilano S.R., Lee D.H., Fukuhara P.S., Chwa M., Nesburn A.B., Udar N., Kenney M.C. (2019). Corneal Oxidative Damage in Keratoconus Cells due to Decreased Oxidant Elimination from Modified Expression Levels of SOD Enzymes, PRDX6, SCARA3, CPSF3, and FOXM1. J. Ophthalmic Vis. Res..

[44] Udar N., Atilano S.R., Brown D.J., Holguin B., Small K., Nesburn A.B., Kenney M.C. (2006). SOD1: A candidate gene for keratoconus. Investig. Ophthalmol. Vis. Sci..

[45] Udar N., Atilano S.R., Small K., Nesburn A.B., Kenney M.C. (2009). SOD1 haplotypes in familial keratoconus. Cornea.

[46] Moschos M.M., Kokolakis N., Gazouli M., Chatziralli I.P., Droutsas D., Anagnou N.P., Ladas I.D. (2015). Polymorphism Analysis of VSX1 and SOD1 Genes in Greek Patients with Keratoconus. Ophthalmic Genet..

[47] Sarker-Nag A., Hutcheon A.E., Karamichos D. (2016). Mitochondrial Profile and Responses to TGF-beta Ligands in Keratoconus. Curr. Eye Res..

[48] McKay T.B., Lyon D., Sarker-Nag A., Priyadarsini S., Asara J.M., Karamichos D. (2015). Quercetin attenuates lactate production and extracellular matrix secretion in keratoconus. Sci. Rep..

[49] Carito V., Bonuccelli G., Martinez-Outschoorn U.E., Whitaker-Menezes D., Caroleo M.C., Cione E., Howell A., Pestell R.G., Lisanti M.P., Sotgia F. (2012). Metabolic remodeling of the tumor microenvironment: Migration stimulating factor (MSF) reprograms myofibroblasts toward lactate production, fueling anabolic tumor growth. Cell Cycle.

[50] Guido C., Whitaker-Menezes D., Lin Z., Pestell R.G., Howell A., Zimmers T.A., Casimiro M.C., Aquila S., Ando S., Martinez-Outschoorn U.E. (2012). Mitochondrial fission induces glycolytic reprogramming in cancer-associated myofibroblasts, driving stromal lactate production, and early tumor growth. Oncotarget.

[51] Phipps R.P., Sime P.J., Xie N., Tan Z., Banerjee S., Cui H., Ge J., Liu R.M., Bernard K., Thannickal V.J. (2015). Glycolytic Reprogramming in Myofibroblast Differentiation and Lung Fibrosis. Am. J. Respir. Crit. Care Med..

[52] Kottmann R.M., Trawick E., Judge J.L., Wahl L.A., Epa A.P., Owens K.M., Thatcher T.H. (2015). Pharmacologic inhibition of lactate production prevents myofibroblast differentiation. Am. J. Physiol. Lung Cell. Mol. Physiol..

[53] McKay T.B., Karamichos D. (2017). Quercetin and the ocular surface: What we know and where we are going. Exp. Biol. Med..

[54] Ertan A., Muftuoglu O. (2008). Keratoconus clinical findings according to different age and gender groups. Cornea.

[55] Fink B.A., Sinnott L.T., Wagner H., Friedman C., Zadnik K. (2010). The influence of gender and hormone status on the severity and progression of keratoconus. Cornea.

[56] Compagnone N.A., Mellon S.H. (1998). Dehydroepiandrosterone: A potential signalling molecule for neocortical organization during development. Proc. Natl. Acad. Sci. USA.

[57] Parker C.R. (1999). Dehydroepiandrosterone and dehydroepiandrosterone sulfate production in the human adrenal during development and aging. Steroids.

[58] McKay T.B., Hjortdal J., Sejersen H., Karamichos D. (2017). Differential Effects of Hormones on Cellular Metabolism in Keratoconus In Vitro. Sci. Rep..

[59] Coco G., Kheirkhah A., Foulsham W., Dana R., Ciolino J.B. (2019). Keratoconus progression associated with hormone replacement therapy. Am. J. Ophthalmol. Case Rep..

[60] Yuksel E., Yalinbas D., Aydin B., Bilgihan K. (2016). Keratoconus Progression Induced by In Vitro Fertilization Treatment. J. Refract. Surg..

[61] Ayan B., Yuksel N., Carhan A., Gumuskaya Ocal B., Akcay E., Cagil N., Asik M.D. (2019). Evaluation estrogen, progesteron and androgen receptor expressions in corneal epithelium in keratoconus. Contact Lens Anterior Eye.

[62] Thanos S., Oellers P., Meyer Zu Horste M., Prokosch V., Schlatt S., Seitz B., Gatzioufas Z. (2016). Role of Thyroxine in the Development of Keratoconus. Cornea.

[63] Priyadarsini S., Hjortdal J., Sarker-Nag A., Sejersen H., Asara J.M., Karamichos D. (2014). Gross cystic disease fluid protein-15/prolactin-inducible protein as a biomarker for keratoconus disease. PLoS ONE.

[64] Stachon T., Stachon A., Hartmann U., Seitz B., Langenbucher A., Szentmary N. (2017). Urea, Uric Acid, Prolactin and fT4 Concentrations in Aqueous Humor of Keratoconus Patients. Curr. Eye Res..

[65] Ljubimov A.V. (2017). Diabetic complications in the cornea. Vis. Res..

[66] Bikbova G., Oshitari T., Tawada A., Yamamoto S. (2012). Corneal changes in diabetes mellitus. Curr. Diabetes Rev..

[67] Markoulli M., Flanagan J., Tummanapalli S.S., Wu J., Willcox M. (2018). The impact of diabetes on corneal nerve morphology and ocular surface integrity. Ocul. Surf..

[68] Vieira-Potter V.J., Karamichos D., Lee D.J. (2016). Ocular Complications of Diabetes and Therapeutic Approaches. BioMed Res. Int..

[69] Dogru M., Katakami C., Inoue M. (2001). Tear function and ocular surface changes in noninsulin-dependent diabetes mellitus. Ophthalmology.

[70] Miller D.D., Hasan S.A., Simmons N.L., Stewart M.W. (2019). Recurrent corneal erosion: A comprehensive review. Clin. Ophthalmol..

[71] Inoue K., Okugawa K., Amano S., Oshika T., Takamura E., Egami F., Umizu G., Aikawa K., Kato S. (2005). Blinking and superficial punctate keratopathy in patients with diabetes mellitus. Eye.

[72] Chang P.-Y., Carrel H., Huang J.-S., Wang I.J., Hou Y.-C., Chen W.-L., Wang J.-Y., Hu F.-R. (2006). Decreased Density of Corneal Basal Epithelium and Subbasal Corneal Nerve Bundle Changes in Patients with Diabetic Retinopathy. Am. J. Ophthalmol..

[73] Asghar O., Petropoulos I.N., Alam U., Jones W., Jeziorska M., Marshall A., Ponirakis G., Fadavi H., Boulton A.J., Tavakoli M. (2014). Corneal confocal microscopy detects neuropathy in subjects with impaired glucose tolerance. Diabetes Care.

[74] Roszkowska A.M., Tringali C.G., Colosi P., Squeri C.A., Ferreri G. (1999). Corneal endothelium evaluation in type I and type II diabetes mellitus. Ophthalmologica.

[75] Priyadarsini S., Sarker-Nag A., Rowsey T.G., Ma J.X., Karamichos D. (2016). Establishment of a 3D In Vitro Model to Accelerate the Development of Human Therapies against Corneal Diabetes. PLoS ONE.

[76] Dehghani C., Pritchard N., Edwards K., Russell A.W., Malik R.A., Efron N. (2016). Abnormal Anterior Corneal Morphology in Diabetes Observed Using In Vivo Laser-scanning Confocal Microscopy. Ocul. Surf..

[77] Saghizadeh M., Kramerov A.A., Yu F.S., Castro M.G., Ljubimov A.V. (2010). Normalization of wound healing and diabetic markers in organ cultured human diabetic corneas by adenoviral delivery of c-Met gene. Investig. Ophthalmol. Vis. Sci..

[78] Saghizadeh M., Epifantseva I., Hemmati D.M., Ghiam C.A., Brunken W.J., Ljubimov A.V. (2013). Enhanced wound healing, kinase and stem cell marker expression in diabetic organ-cultured human corneas upon MMP-10 and cathepsin F gene silencing. Investig. Ophthalmol. Vis. Sci..

[79] Bao F., Deng M., Zheng X., Li L., Zhao Y., Cao S., Yu A., Wang Q., Huang J., Elsheikh A. (2017). Effects of diabetes mellitus on biomechanical properties of the rabbit cornea. Exp. Eye Res..

[80] Rosenberg M.E., Tervo T.M., Immonen I.J., Muller L.J., Gronhagen-Riska C., Vesaluoma M.H. (2000). Corneal structure and sensitivity in type 1 diabetes mellitus. Investig. Ophthalmol. Vis. Sci..

[81] Kumar N., Pop-Busui R., Musch D.C., Reed D.M., Momont A.C., Hussain M., Raval N., Moroi S.E., Shtein R. (2018). Central Corneal Thickness Increase Due to Stromal Thickening With Diabetic Peripheral Neuropathy Severity. Cornea.

[82] Scheler A., Spoerl E., Boehm A.G. (2012). Effect of diabetes mellitus on corneal biomechanics and measurement of intraocular pressure. Acta Ophthalmol..

[83] Hager A., Wegscheider K., Wiegand W. (2009). Changes of extracellular matrix of the cornea in diabetes mellitus. Graefes Arch. Clin. Exp. Ophthalmol..

[84] Kotecha A., Oddone F., Sinapis C., Elsheikh A., Sinapis D., Sinapis A., Garway-Heath D.F. (2010). Corneal biomechanical characteristics in patients with diabetes mellitus. J. Cataract. Refract. Surg..

[85] Goldich Y., Barkana Y., Gerber Y., Rasko A., Morad Y., Harstein M., Avni I., Zadok D. (2009). Effect of diabetes mellitus on biomechanical parameters of the cornea. J. Cataract. Refract. Surg..

[86] Sahin A., Bayer A., Ozge G., Mumcuoglu T. (2009). Corneal biomechanical changes in diabetes mellitus and their influence on intraocular pressure measurements. Investig. Ophthalmol. Vis. Sci..

[87] Perez-Rico C., Gutierrez-Ortiz C., Gonzalez-Mesa A., Zandueta A.M., Moreno-Salgueiro A., Germain F. (2015). Effect of diabetes mellitus on Corvis ST measurement process. Acta Ophthalmol..

[88] Beato J.N., Esteves-Leandro J., Reis D., Falcao M., Rosas V., Carneiro Â., Falcão R. (2019). Structural and Biomechanical Corneal Differences between Type 2 Diabetic and Nondiabetic Patients. J. Ophthalmol..

[89] Ramm L., Herber R., Spoerl E., Pillunat L.E., Terai N. (2019). Measurement of Corneal Biomechanical Properties in Diabetes Mellitus Using the Ocular Response Analyzer and the Corvis ST. Cornea.

[90] Monnier V.M., Cerami A. (1981). Nonenzymatic browning in vivo: Possible process for aging of long-lived proteins. Science.

[91] Monnier V.M., Kohn R.R., Cerami A. (1984). Accelerated age-related browning of human collagen in diabetes mellitus. Proc. Natl. Acad. Sci. USA.

[92] Li Y., Fessel G., Georgiadis M., Snedeker J.G. (2013). Advanced glycation end-products diminish tendon collagen fiber sliding. Matrix Biol..

[93] Andreassen T.T., Seyer-Hansen K., Bailey A.J. (1981). Thermal stability, mechanical properties and reducible cross-links of rat tail tendon in experimental diabetes. Biochim. Biophys. Acta.

[94] Kaji Y., Usui T., Oshika T., Matsubara M., Yamashita H., Araie M., Murata T., Ishibashi T., Nagai R., Horiuchi S. (2000). Advanced glycation end products in diabetic corneas. Investig. Ophthalmol. Vis. Sci..

[95] Bai P., Phua K., Hardt T., Cernadas M., Brodsky B. (1992). Glycation alters collagen fibril organization. Connect. Tissue Res..

[96] Singh R., Barden A., Mori T., Beilin L. (2001). Advanced glycation end-products: A review. Diabetologia.

[97] Azar D.T., Spurr-Michaud S.J., Tisdale A.S., Gipson I.K. (1989). Decreased Penetration of Anchoring Fibrils Into the Diabetic Stroma: A Morphometric Analysis. Arch. Ophthalmol..

[98] Griffith M., Osborne R., Munger R., Xiong X., Doillon C.J., Laycock N.L., Hakim M., Song Y., Watsky M.A. (1999). Functional human corneal equivalents constructed from cell lines. Science.

[99] Karamichos D., Zareian R., Guo X., Hutcheon A.E., Ruberti J.W., Zieske J.D. (2012). Novel in Vitro Model for Keratoconus Disease. J. Funct. Biomater..

[100] Bykhovskaya Y., Li X., Epifantseva I., Haritunians T., Siscovick D., Aldave A., Szczotka-Flynn L., Iyengar S.K., Taylor K.D., Rotter J.I. (2012). Variation in the lysyl oxidase (LOX) gene is associated with keratoconus in family-based and case-control studies. Investig. Ophthalmol. Vis. Sci..

[101] Dudakova L., Liskova P., Trojek T., Palos M., Kalasova S., Jirsova K. (2012). Changes in lysyl oxidase (LOX) distribution and its decreased activity in keratoconus corneas. Exp. Eye Res..

[102] Shetty R., Sathyanarayanamoorthy A., Ramachandra R.A., Arora V., Ghosh A., Srivatsa P.R., Pahuja N., Nuijts R.M., Sinha-Roy A., Mohan R.R. (2015). Attenuation of lysyl oxidase and collagen gene expression in keratoconus patient corneal epithelium corresponds to disease severity. Mol. Vis..

[103] De Bonis P., Laborante A., Pizzicoli C., Stallone R., Barbano R., Longo C., Mazzilli E., Zelante L., Bisceglia L. (2011). Mutational screening of VSX1, SPARC, SOD1, LOX, and TIMP3 in keratoconus. Mol. Vis..

[104] Cho K.J., Mok J.W., Choi M.Y., Kim J.Y., Joo C.K. (2013). Changes in corneal sensation and ocular surface in patients with asymmetrical keratoconus. Cornea.

[105] Dogru M., Karakaya H., Ozcetin H., Erturk H., Yucel A., Ozmen A., Baykara M., Tsubota K. (2003). Tear function and ocular surface changes in keratoconus. Ophthalmology.

[106] Kenney M.C., Chwa M., Opbroek A.J., Brown D.J. (1994). Increased gelatinolytic activity in keratoconus keratocyte cultures. A correlation to an altered matrix metalloproteinase-2/tissue inhibitor of metalloproteinase ratio. Cornea.

[107] Brown D., Chwa M.M., Opbroek A., Kenney M.C. (1993). Keratoconus corneas: Increased gelatinolytic activity appears after modification of inhibitors. Curr. Eye Res..

[108] di Martino E., Ali M., Inglehearn C.F. (2019). Matrix metalloproteinases in keratoconus—Too much of a good thing?. Exp. Eye Res..

[109] Arbab M., Tahir S., Niazi M.K., Ishaq M., Hussain A., Siddique P.M., Saeed S., Khan W.A., Qamar R., Butt A.M. (2017). TNF-alpha Genetic Predisposition and Higher Expression of Inflammatory Pathway Components in Keratoconus. Investig. Ophthalmol. Vis. Sci..

[110] Pahuja N., Kumar N.R., Shroff R., Shetty R., Nuijts R.M., Ghosh A., Sinha-Roy A., Chaurasia S.S., Mohan R.R., Ghosh A. (2016). Differential Molecular Expression of Extracellular Matrix and Inflammatory Genes at the Corneal Cone Apex Drives Focal Weakening in Keratoconus. Investig. Ophthalmol. Vis. Sci..

[111] Wisse R.P., Kuiper J.J., Gans R., Imhof S., Radstake T.R., Van der Lelij A. (2015). Cytokine Expression in Keratoconus and its Corneal Microenvironment: A Systematic Review. Ocul. Surf..

[112] Foster J.W., Shinde V., Soiberman U.S., Sathe G., Liu S., Wan J., Qian J., Dauoud Y., Pandey A., Jun A.S. (2018). Integrated Stress Response and Decreased ECM in Cultured Stromal Cells From Keratoconus Corneas. Investig. Ophthalmol. Vis. Sci..

[113] Mas Tur V., MacGregor C., Jayaswal R., O’Brart D., Maycock N. (2017). A review of keratoconus: Diagnosis, pathophysiology, and genetics. Surv. Ophthalmol..

[114] Luz A., Lopes B., Hallahan K.M., Valbon B., Ramos I., Faria-Correia F., Schor P., Dupps W.J., Ambrosio R. (2016). Enhanced Combined Tomography and Biomechanics Data for Distinguishing Forme Fruste Keratoconus. J. Refract. Surg..

[115] Ahmadi Hosseini S.M., Abolbashari F., Niyazmand H., Sedaghat M.R. (2014). Efficacy of corneal tomography parameters and biomechanical characteristic in keratoconus detection. Cont Lens Anterior Eye.

[116] Ayar O., Ozmen M.C., Muftuoglu O., Akdemir M.O., Koc M., Ozulken K. (2015). In-vivo corneal biomechanical analysis of unilateral keratoconus. Int. J. Ophthalmol..

[117] Mikula E.R., Jester J.V., Juhasz T. (2016). Measurement of an Elasticity Map in the Human Cornea Measurement of Elasticity Map in Human Cornea. Investig. Ophthalmol. Vis. Sci..

[118] Mikula E., Winkler M., Juhasz T., Brown D.J., Shoa G., Tran S., Kenney M.C., Jester J.V. (2018). Axial mechanical and structural characterization of keratoconus corneas. Exp. Eye Res..

[119] Andreassen T.T., Hjorth Simonsen A., Oxlund H. (1980). Biomechanical properties of keratoconus and normal corneas. Exp. Eye Res..

[120] Kenney M.C., Nesburn A.B., Burgeson R.E., Butkowski R.J., Ljubimov A.V. (1997). Abnormalities of the extracellular matrix in keratoconus corneas. Cornea.

[121] Meek K.M., Tuft S.J., Huang Y., Gill P.S., Hayes S., Newton R.H., Bron A.J. (2005). Changes in collagen orientation and distribution in keratoconus corneas. Investig. Ophthalmol. Vis. Sci..

[122] Daxer A., Fratzl P. (1997). Collagen fibril orientation in the human corneal stroma and its implication in keratoconus. Investig. Ophthalmol. Vis. Sci..

[123] Mathew J.H., Goosey J.D., Soderberg P.G., Bergmanson J.P. (2015). Lamellar changes in the keratoconic cornea. Acta Ophthalmol..

[124] Tian L., Ko M.W., Wang L.K., Zhang J.Y., Li T.J., Huang Y.F., Zheng Y.P. (2014). Assessment of ocular biomechanics using dynamic ultra high-speed Scheimpflug imaging in keratoconic and normal eyes. J. Refract. Surg..

[125] Pena-Garcia P., Peris-Martinez C., Abbouda A., Ruiz-Moreno J.M. (2016). Detection of subclinical keratoconus through non-contact tonometry and the use of discriminant biomechanical functions. J. Biomech..

[126] Esser N., Legrand-Poels S., Piette J., Scheen A.J., Paquot N. (2014). Inflammation as a link between obesity, metabolic syndrome and type 2 diabetes. Diabetes Res. Clin. Pract..

[127] Shajari M., Eberhardt E., Muller M., Al Khateeb G., Friderich S., Remy M., Kohnen T. (2016). Effects of Atopic Syndrome on Keratoconus. Cornea.

[128] Rahi A., Davies P., Ruben M., Lobascher D., Menon J. (1977). Keratoconus and coexisting atopic disease. Br. J. Ophthalmol..

[129] Skaaby T., Husemoen L.L., Thuesen B.H., Jeppesen J., Linneberg A. (2015). The association of atopy with incidence of ischemic heart disease, stroke, and diabetes. Endocrine.

[130] Jasser-Nitsche H., Varga E.M., Borkenstein H.M., Hontzsch J., Suppan E., Weinhandl G., Pieringer L., Avian A., Frohlich-Reiterer E. (2017). Type 1 diabetes in children and adolescents is not associated with a reduced prevalence of atopy and allergic diseases. Pediatr. Diabetes.

[131] Seiler T., Huhle S., Spoerl E., Kunath H. (2000). Manifest diabetes and keratoconus: A retrospective case-control study. Graefes Arch. Clin. Exp. Ophthalmol..

[132] Kuo I.C., Broman A., Pirouzmanesh A., Melia M. (2006). Is there an association between diabetes and keratoconus?. Ophthalmology.

[133] Naderan M., Naderan M., Rezagholizadeh F., Zolfaghari M., Pahlevani R., Rajabi M.T. (2014). Association between diabetes and keratoconus: A case-control study. Cornea.

[134] Woodward M.A., Blachley T.S., Stein J.D. (2016). The Association Between Sociodemographic Factors, Common Systemic Diseases, and Keratoconus: An Analysis of a Nationwide Heath Care Claims Database. Ophthalmology.

[135] Kosker M., Suri K., Hammersmith K.M., Nassef A.H., Nagra P.K., Rapuano C.J. (2014). Another look at the association between diabetes and keratoconus. Cornea.

[136] Vatankhah N., Jahangiri Y., Landry G.J., Moneta G.L., Azarbal A.F. (2017). Effect of systemic insulin treatment on diabetic wound healing. Wound Repair Regen..

[137] Lima M.H., Caricilli A.M., de Abreu L.L., Araujo E.P., Pelegrinelli F.F., Thirone A.C., Tsukumo D.M., Pessoa A.F., dos Santos M.F., de Moraes M.A. (2012). Topical insulin accelerates wound healing in diabetes by enhancing the AKT and ERK pathways: A double-blind placebo-controlled clinical trial. PLoS ONE.

[138] Chen D.K., Frizzi K.E., Guernsey L.S., Ladt K., Mizisin A.P., Calcutt N.A. (2013). Repeated monitoring of corneal nerves by confocal microscopy as an index of peripheral neuropathy in type-1 diabetic rodents and the effects of topical insulin. J. Peripher. Nerv. Syst..

[139] Zagon I.S., Klocek M.S., Sassani J.W., McLaughlin P.J. (2007). Use of topical insulin to normalize corneal epithelial healing in diabetes mellitus. Arch. Ophthalmol..

[140] Fai S., Ahem A., Mustapha M., Mohd Noh U.K., Bastion M.C. (2017). Randomized Controlled Trial of Topical Insulin for Healing Corneal Epithelial Defects Induced During Vitreoretinal Surgery in Diabetics. Asia Pac. J. Ophthalmol..

[141] Immonen J.A., Zagon I.S., McLaughlin P.J. (2014). Selective blockade of the OGF-OGFr pathway by naltrexone accelerates fibroblast proliferation and wound healing. Exp. Biol. Med..

[142] Zagon I.S., Klocek M.S., Sassani J.W., Mauger D.T., McLaughlin P.J. (2006). Corneal safety of topically applied naltrexone. J. Ocul. Pharmacol..

[143] Klocek M.S., Sassani J.W., McLaughlin P.J., Zagon I.S. (2009). Naltrexone and insulin are independently effective but not additive in accelerating corneal epithelial healing in type I diabetic rats. Exp. Eye Res..

[144] Kador P.F., Wyman M., Oates P.J. (2016). Aldose reductase, ocular diabetic complications and the development of topical Kinostat((R)). Prog. Retin. Eye Res..

[145] Jacot J.L., Hosotani H., Glover J.P., Lois N., Robison W.G. (1998). Diabetic-like corneal sensitivity loss in galactose-fed rats ameliorated with aldose reductase inhibitors. J. Ocul. Pharmacol..

[146] Kubo E., Mori K., Kobayashi T., Takahashi Y., Yokoi N., Kinoshita S., Kasahara T., Yonezawa H., Akagi Y. (1998). Effect of aldose reductase inhibitor on corneal epithelial barrier function in galactose-fed dogs. J. Ocul. Pharmacol..

[147] Guo C., Li M., Qi X., Lin G., Cui F., Li F., Wu X. (2016). Intranasal delivery of nanomicelle curcumin promotes corneal epithelial wound healing in streptozotocin-induced diabetic mice. Sci. Rep..

[148] He J., Pham T.L., Kakazu A., Bazan H.E.P. (2017). Recovery of Corneal Sensitivity and Increase in Nerve Density and Wound Healing in Diabetic Mice After PEDF Plus DHA Treatment. Diabetes.

[149] O’Brart D.P.S. (2017). Corneal collagen crosslinking for corneal ectasias: A review. Eur. J. Ophthalmol..

[150] Brierly S.C., Izquierdo L., Mannis M.J. (2000). Penetrating keratoplasty for keratoconus. Cornea.

[151] Lim L., Pesudovs K., Coster D.J. (2000). Penetrating keratoplasty for keratoconus: Visual outcome and success. Ophthalmology.

[152] Sharif K., Casey T. (1991). Penetrating keratoplasty for keratoconus: Complications and long-term success. Br. J. Ophthalmol..

[153] Patel S.V., Malta J.B., Banitt M.R., Mian S.I., Sugar A., Elner V.M., Tester R., Farjo Q., Soong H.K. (2009). Recurrent ectasia in corneal grafts and outcomes of repeat keratoplasty for keratoconus. Br. J. Ophthalmol..

[154] Kremer I., Eagle R.C., Rapuano C.J., Laibson P.R. (1995). Histologic evidence of recurrent keratoconus seven years after keratoplasty. Am. J. Ophthalmol..

[155] Spoerl E., Huhle M., Seiler T. (1998). Induction of cross-links in corneal tissue. Exp. Eye Res..

[156] Sporl E., Huhle M., Kasper M., Seiler T. (1997). Increased rigidity of the cornea caused by intrastromal cross-linking. Ophthalmologe.

[157] Godefrooij D.A., Gans R., Imhof S.M., Wisse R.P. (2016). Nationwide reduction in the number of corneal transplantations for keratoconus following the implementation of cross-linking. Acta Ophthalmol..

[158] Wollensak G., Spoerl E., Seiler T. (2003). Riboflavin/ultraviolet-a-induced collagen crosslinking for the treatment of keratoconus. Am. J. Ophthalmol..

[159] Kymionis G.D., Kontadakis G.A., Hashemi K.K. (2017). Accelerated versus conventional corneal crosslinking for refractive instability: An update. Curr. Opin. Ophthalmol..

[160] Thavarajah R., Mudimbaimannar V.K., Elizabeth J., Rao U.K., Ranganathan K. (2012). Chemical and physical basics of routine formaldehyde fixation. J. Oral Maxillofac. Pathol..

[161] McGregor D., Bolt H., Cogliano V., Richter-Reichhelm H.B. (2006). Formaldehyde and glutaraldehyde and nasal cytotoxicity: Case study within the context of the 2006 IPCS Human Framework for the Analysis of a cancer mode of action for humans. Crit. Rev. Toxicol..

[162] Mattson G., Conklin E., Desai S., Nielander G., Savage M.D., Morgensen S. (1993). A practical approach to crosslinking. Mol. Biol. Rep..

[163] Viguet-Carrin S., Garnero P., Delmas P. (2006). The role of collagen in bone strength. Osteoporos. Int..

[164] Szauter K.M., Cao T., Boyd C.D., Csiszar K. (2005). Lysyl oxidase in development, aging and pathologies of the skin. Pathol. Biol..

[165] Kagan H.M., Li W. (2003). Lysyl oxidase: Properties, specificity, and biological roles inside and outside of the cell. J. Cell. Biochem..

[166] Takaoka A., Babar N., Hogan J., Kim M., Price M.O., Price F.W., Trokel S.L., Paik D.C. (2016). An Evaluation of Lysyl Oxidase-Derived Cross-Linking in Keratoconus by Liquid Chromatography/Mass Spectrometry. Investig. Ophthalmol. Vis. Sci..

[167] Yamauchi M., Chandler G.S., Tanzawa H., Katz E.P. (1996). Cross-linking and the molecular packing of corneal collagen. Biochem. Biophys. Res. Commun..

[168] Erler J.T., Bennewith K.L., Nicolau M., Dornhöfer N., Kong C., Le Q.-T., Chi J.-T.A., Jeffrey S.S., Giaccia A.J. (2006). Lysyl oxidase is essential for hypoxia-induced metastasis. Nature.

[169] Levental K.R., Yu H., Kass L., Lakins J.N., Egeblad M., Erler J.T., Fong S.F.T., Csiszar K., Giaccia A., Weninger W. (2009). Matrix Crosslinking Forces Tumor Progression by Enhancing Integrin Signaling. Cell.

[170] Akiri G., Sabo E., Dafni H., Vadasz Z., Kartvelishvily Y., Gan N., Kessler O., Cohen T., Resnick M., Neeman M. (2003). Lysyl oxidase-related protein-1 promotes tumor fibrosis and tumor progression in vivo. Cancer Res..

[171] di Ferrante N., Leachman R., Angelini P., Donnelly P.V., Francis G., Almazan A., Segni G. (1975). Lysyl oxidase deficiency in Ehlers–Danlos syndrome type V. Connect. Tissue Res..

[172] Royce P., Camakaris J., Danks D. (1980). Reduced lysyl oxidase activity in skin fibroblasts from patients with Menkes’ syndrome. Biochem. J..

[173] Kagan H.M., Trackman P.C. (1991). Properties and function of lysyl oxidase. Am. J. Respir. Cell Mol. Biol..

[174] Murray J.C. (1994). Keloids and hypertrophic scars. Clin. Dermatol..

[175] Robins S.P. (2007). Biochemistry and functional significance of collagen cross-linking. Biochem. Soc. Trans..

[176] Maillard L. (1912). Action of amino acids on sugars. Formation of melanoidins in a methodical way. C. R. Acad. Sci..

[177] Rohlfing C.L., Wiedmeyer H.-M., Little R.R., England J.D., Tennill A., Goldstein D.E. (2002). Defining the relationship between plasma glucose and HbA1c: Analysis of glucose profiles and HbA1c in the Diabetes Control and Complications Trial. Diabetes Care.

[178] Sady C., Khosrof S., Nagaraj R. (1995). Advanced Maillard Reaction and Crosslinking of Corneal Collagen in Diabetes. Biochem. Biophys. Res. Commun..

[179] Sell D.R., Monnier V.M. (1989). Structure elucidation of a senescence cross-link from human extracellular matrix. Implication of pentoses in the aging process. J. Biol. Chem..

[180] McCall A.S., Kraft S., Edelhauser H.F., Kidder G.W., Lundquist R.R., Bradshaw H.E., Dedeic Z., Dionne M.J.C., Clement E.M., Conrad G.W. (2010). Mechanisms of Corneal Tissue Cross-linking in Response to Treatment with Topical Riboflavin and Long-Wavelength Ultraviolet Radiation (UVA). Investig. Ophthalmol. Vis. Sci..

[181] Raiskup F., Spoerl E. (2013). Corneal crosslinking with riboflavin and ultraviolet A. I. Principles. Ocul. Surf..

[182] Zhang Y., Conrad A.H., Conrad G.W. (2011). Effects of ultraviolet-A and riboflavin on the interaction of collagen and proteoglycans during corneal cross-linking. J. Biol. Chem..

[183] Kato Y., Uchida K., Kawakishi S. (1994). Aggregation of collagen exposed to UVA in the presence of riboflavin: A plausible role of tyrosine modification. Photochem. Photobiol..

[184] Wollensak G. (2006). Crosslinking treatment of progressive keratoconus: New hope. Curr. Opin. Ophthalmol..

[185] Spoerl E., Wollensak G., Seiler T. (2004). Increased resistance of crosslinked cornea against enzymatic digestion. Curr. Eye Res..

[186] Kymionis G.D., Grentzelos M.A., Kounis G.A., Portaliou D.M., Detorakis E.T., Magarakis M., Karampatakis V.E., Pallikaris I.G. (2010). Intraocular pressure measurements after corneal collagen crosslinking with riboflavin and ultraviolet A in eyes with keratoconus. J. Cataract. Refract. Surg..

[187] Romppainen T., Bachmann L.M., Kaufmann C., Kniestedt C., Mrochen M., Thiel M.A. (2007). Effect of riboflavin-UVA–induced collagen cross-linking on intraocular pressure measurement. Investig. Ophthalmol. Vis. Sci..

[188] Wollensak G., Wilsch M., Spoerl E., Seiler T. (2004). Collagen fiber diameter in the rabbit cornea after collagen crosslinking by riboflavin/UVA. Cornea.

[189] Wollensak G., Spoerl E., Seiler T. (2003). Stress-strain measurements of human and porcine corneas after riboflavin-ultraviolet-A-induced cross-linking. J. Cataract. Refract. Surg..

[190] Wollensak G., Iomdina E. (2009). Long-term biomechanical properties of rabbit cornea after photodynamic collagen crosslinking. Acta Ophthalmol..

[191] Bradford S.M., Mikula E.R., Juhasz T., Brown D.J., Jester J.V. (2018). Collagen fiber crimping following in vivo UVA-induced corneal crosslinking. Exp. Eye Res..

[192] Hersh P.S., Stulting R.D., Muller D., Durrie D.S., Rajpal R.K. (2017). United States Multicenter Clinical Trial of Corneal Collagen Crosslinking for Keratoconus Treatment. Ophthalmology.

[193] Hersh P.S., Stulting R.D., Muller D., Durrie D.S., Rajpal R.K. (2017). U.S. Multicenter Clinical Trial of Corneal Collagen Crosslinking for Treatment of Corneal Ectasia after Refractive Surgery. Ophthalmology.

[194] Reynaert N.L., Gopal P., Rutten E.P.A., Wouters E.F.M., Schalkwijk C.G. (2016). Advanced glycation end products and their receptor in age-related, non-communicable chronic inflammatory diseases; Overview of clinical evidence and potential contributions to disease. Int. J. Biochem. Cell Biol..

[195] Wollensak G., Spoerl E., Reber F., Seiler T. (2004). Keratocyte cytotoxicity of riboflavin/UVA-treatment in vitro. Eye.

[196] Mazzotta C., Hafezi F., Kymionis G., Caragiuli S., Jacob S., Traversi C., Barabino S., Randleman J.B. (2015). In Vivo Confocal Microscopy after Corneal Collagen Crosslinking. Ocul. Surf..

[197] Jordan C., Patel D.V., Abeysekera N., McGhee C.N. (2014). In vivo confocal microscopy analyses of corneal microstructural changes in a prospective study of collagen cross-linking in keratoconus. Ophthalmology.

[198] Wollensak G., Sporl E., Reber F., Pillunat L., Funk R. (2003). Corneal endothelial cytotoxicity of riboflavin/UVA treatment in vitro. Ophthalmic Res..

[199] Sharif R., Hjortdal J., Sejersen H., Frank G., Karamichos D. (2017). Human in vitro Model Reveals the Effects of Collagen Cross-linking on Keratoconus Pathogenesis. Sci. Rep..

[200] Sharif R., Fowler B., Karamichos D. (2018). Collagen cross-linking impact on keratoconus extracellular matrix. PLoS ONE.

[201] Vallabh N.A., Romano V., Willoughby C.E. (2017). Mitochondrial dysfunction and oxidative stress in corneal disease. Mitochondrion.

[202] Ashoori M., Saedisomeolia A. (2014). Riboflavin (vitamin B(2)) and oxidative stress: A review. Br. J. Nutr..

[203] Lynnerup N., Kjeldsen H., Heegaard S., Jacobsen C., Heinemeier J. (2008). Radiocarbon dating of the human eye lens crystallines reveal proteins without carbon turnover throughout life. PLoS ONE.

[204] Truscott R.J.W., Friedrich M.G. (2016). The etiology of human age-related cataract. Proteins don’t last forever. Biochim. Biophys. Acta.

